# PCP Pincer Complexes of Titanium in the +3 and +4 Oxidation States

**DOI:** 10.1021/acs.organomet.2c00662

**Published:** 2023-03-13

**Authors:** Benedek Stadler, Hilary H. Y. Meng, Sara Belazregue, Leah Webster, Alberto Collauto, Keelan M. Byrne, Tobias Krämer, F. Mark Chadwick

**Affiliations:** † Molecular Sciences Research Hub, Department of Chemistry, Imperial College London, 82 Wood Lane, London W12 0BZ, United Kingdom; ‡ Department of Chemistry, Maynooth University, Maynooth, Co. Kildare W23 F2H6, Ireland

## Abstract

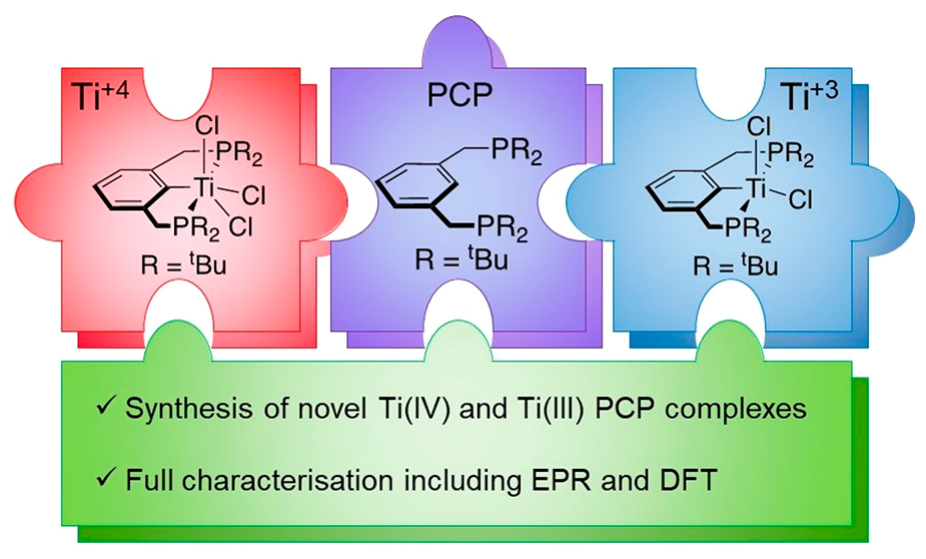

Ti(IV) and Ti(III) complexes using the ^tBu^PCP ligand have been synthesized (^tBu^PCP = C_6_H_3_-2,6-(CH_2_P^t^Bu_2_)_2_). The [^tBu^PCP]Li synthon can be reacted with TiCl_4_(THF)_2_ to form (^tBu^PCP)TiCl_3_ (**1**) in limited yields due to significant reduction of the titanium synthon. The Ti(III) complex (^tBu^PCP)TiCl_2_ (**2**) has been further characterized. This can have half an equivalent of halide abstracted to form [{(^tBu^PCP)TiCl}_2_{μ-Cl}][B(C_6_F_5_)_4_] (**3**) and can also be methylated, forming (^tBu^PCP)TiMe_2_ (**4**). All the Ti(III) complexes have been characterized using EPR and X-ray crystallography, giving insight into their electronic structures, which are further supported by DFT calculations.

## Introduction

Since the work of Shaw in 1976, pincer complexes have been a powerful weapon in the organometallic chemist’s arsenal.^1^ Formally defined as tridentate ligands which usually hold a fixed meridional geometry, their high thermal stability and extraordinary tunability has resulted in their use in myriad applications.^2−4^ Pincer complexes have been applied extensively in catalysis, particularly for hydrogenation/dehydrogenation reactions.^5−7^ They have also been applied in the area of small-molecule activation—for example, in the cleavage and reduction of dinitrogen.^8−10^

The bulk of research carried out with pincer complexes has focused on the late transition metals. More recently, work has moved toward more earth-abundant metals, such as iron and manganese.^11,12^ However, there is still a dearth of research on pincer complexes of the early transition metals, particularly those that involve a ligating phosphorus. Previous work using “PNP”-type ligands (where “PNP” represents the three ligating elements) with group 4 metals focused primarily on dinitrogen activation,^13,14^ though other exciting reactivities such as C–H activation by alkylidenes have also been demonstrated.^15,16^ These examples of challenging chemistry highlight the need to further develop the area of early-transition-metal pincer chemistry. Until very recently there were no examples of group 4 “PCP”-type pincer complexes, despite this being the original class developed by Shaw.^1^ We recently synthesized a library of (^R^POCOP)Ti complexes (^R^POCOP = C_6_H_3_-2,6-(OPR_2_)_2_; R = *tert*-butyl, isopropyl) and used this pincer framework to form early-transition-metal hydride complexes.^17^ Others have recently reported (^R^POCOP)Ti(CH_2_SiMe_3_)_2_ and (^R^PCP)Ti(CH_2_SiMe_3_)_2_ (^R^PCP = C_6_H_3_-2,6-(CH_2_PR_2_)_2_; R = *tert*-butyl) complexes as catalysts for styrene polymerization.^18^ Here we build on this work to further explore the (^R^PCP)Ti motif, expanding it to Ti(IV) complexes, as well as present EPR data for known and new Ti(III) complexes.

## Results and Discussion

Very recently the deployment of PCP-type ligands onto early transition metals has been described by us and others.^17−20^ Reacting ^n^BuLi with C_6_H_3_-1-Br-2,6-(CH_2_P^t^Bu_2_)_2_ resulted in the formation of [^tBu^PCP]Li. Others have described forming this *in situ*, but it can be isolated as a highly reactive white solid in 81% yield.^18,21^

**Scheme 1:**
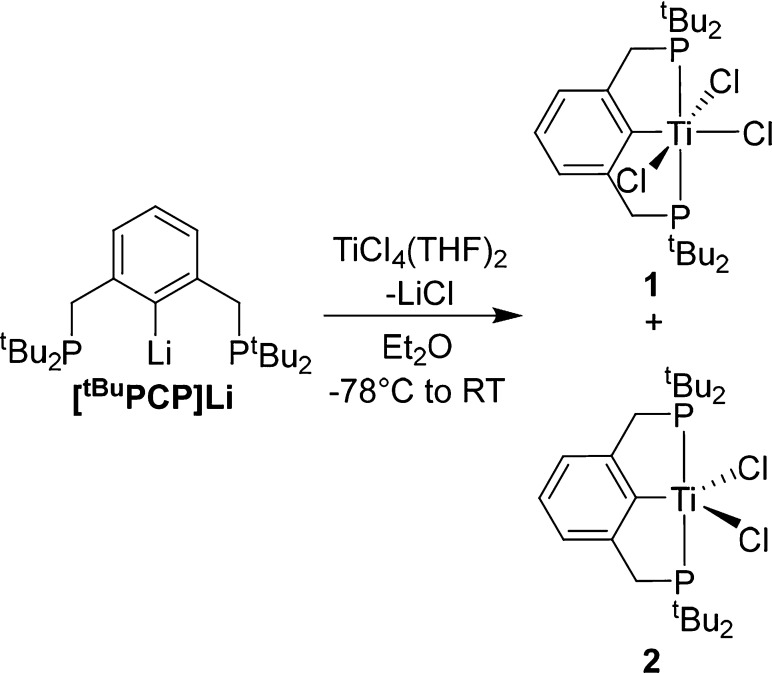
Synthesis of Ti(III) and Ti(IV) PCP Complexes

Targeting the formation of Ti(IV) PCP complexes, [^tBu^PCP]Li was reacted with TiCl_4_(THF)_2_ in Et_2_O, resulting in the immediate formation of a red solution. Upon workup the desired product (^tBu^PCP)TiCl_3_ (**1**) could be isolated in low yield (30%). An *in situ*^1^H NMR study of the reaction demonstrates the reason for this poor yield, with broad resonances being present as well as the resonances attributed to **1** (see Figures S1 and S2). These broad resonances are the result of the reduction of the Ti(IV) center to the Ti(III) complex (^tBu^PCP)TiCl_2_ (**2**; Scheme 1). The extent to which this reduction occurs is highly solvent dependent; the use of THF rather than Et_2_O solely results in Ti(III) products. The synthesis of **1** was attempted in a range of solvents, with none giving better yields than Et_2_O. It should be noted this reduction is somewhat reminiscent of other Ti pincer chemistry—for example, a “PNP”-type Ti(IV) chloride complex could not be alkylated by Grignard reagents without reduction.^22^

**Figure 1:**
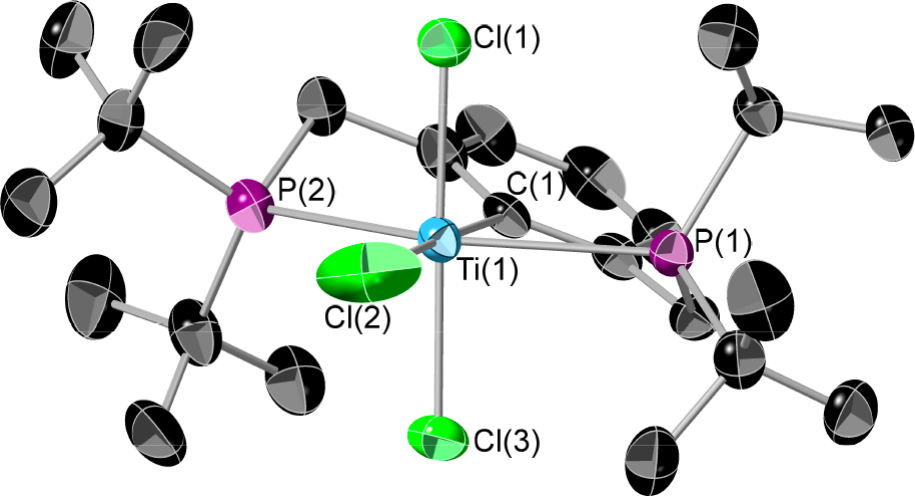
Single-crystal X-ray structure of **1**. Thermal ellipsoids are at 50% probability, and hydrogen atoms are omitted for clarity. Element colors: Ti (sky blue), P (purple), Cl (green), C (black). Selected bond lengths (Å) and angles (deg): Ti(1)–Cl(1) 2.268(2), Ti(1)–Cl(2) 2.318(2), Ti(1)–Cl(3) 2.304(2), Ti(1)–P(1) 2.679(2), Ti(1)–P(2) 2.675(2), Ti(1)–C(1) 2.204(7); Cl(1)–Ti(1)–Cl(2) 92.16(10), Cl(1)–Ti(1)–P(1) 91.17(8), Cl(1)–Ti(1)–P(2) 87.64(8), Cl(1)–Ti(1)–C(1) 89.9(2), Cl(3)–Ti(1)–Cl(2) 90.02(10), Cl(3)–Ti(1)–P(1) 87.95(8), Cl(3)–Ti(1)–P(2) 92.10(8), Cl(3)–Ti(1)–C(1) 88.0(2).

Single crystals of **1** can be grown by cooling a saturated pentane solution. **1** crystallizes as blood red rods with two molecules in the asymmetric unit, one of which is shown in Figure 1. The complexes have almost equivalent structures; thus, only one set of structural parameters is considered. The metal center adopts an almost perfect octahedral geometry with the sums of the angles in the C1–P1–Cl2–P2 plane and C1–Cl1–Cl2–Cl3 plane being 360.1(6) and 360.0(6)°, respectively. This regular structure of **1** is in direct contrast to the equivalent “POCOP” complex C_6_H_3_-2,6-(O_2_P^t^Bu_2_)_2_TiCl_3_, which has an extremely distorted octahedral structure.^17^ In order to probe the electronic structures of these two analogous complexes, DFT calculations were performed.

The optimized geometry of **1** reproduces the crystallographic data with good accuracy (Figure S4), the Ti–P bond lengths are 2.62 and 2.67 Å (vs 2.679(2) and 2.675(2) Å in Figure 1), while the Ti–Cl bonds are 2.29–2.31 Å vs their crystallographic counterparts of 2.268(2), 2.318(2), and 2.304(2) Å. The Ti–C bond distance of 2.21 Å is also close to the crystallographic value (2.204(7) Å). Notably, the C–Ti–Cl angle involving the equatorial Cl ligand is somewhat distorted from linearity (calcd 162.1° vs exptl 173.6°), while the axial Cl–Ti–Cl angle (173.1°) remains close to the experimental value (177.7°). A wider survey of the potential energy surface reveals a second shallow local minimum only 3.1 kcal mol^–1^ above the equilibrium structure, which adopts a strongly distorted structure analogous to that of the ^tBu^POCOP congener. In both of these distorted structures the axial and equatorial C–Ti–Cl angles are significantly shifted off the linear axis (∼145°). This geometry corresponds to the equilibrium structure for (^tBu^POCOP)TiCl_3_, lying energetically ∼8 kcal mol^–1^ below the *C*_2_-symmetric conformer (nonminimum geometry that serves as a common reference point; Figure S4). In contrast, the potential energy landscape of **1** is much flatter (Figure S7), featuring the *C*_2_-symmetric (0.7 kcal mol^–1^) and distorted *C*_1_-symmetric minima (3.1 kcal mol^–1^) close to the ground state. The above observations imply that the aryl and phosphite groups have a stronger donating effect in the ^tBu^POCOP ligand. It is worth noting that the phosphite shows a significantly *shorter* Ti–P bond distance (∼2.60 Å) than the phosphine (∼2.65 Å). Recent studies suggest that phosphites may be stronger net donors toward high-oxidation-state complexes,^23,24^ contrary to the usual ranking found with late transition metals.^25^ Deviations from ideal bond angles in octahedral complexes with low electron counts have been attributed to second-order Jahn–Teller effects,^26−28^ in which a descent in symmetry allows for mixing of occupied (4*p*-based) and vacant (3*d*-based) metal-centered frontier orbitals. The effect is increased overlap with ligand orbitals and energetic stabilization of the resulting bonding hybrid orbital. In the present case the distortion in (^tBu^POCOP)TiCl_3_ results in strengthening of the Ti–C σ-bond between the phenyl ligand and Ti, driven by mixing of the C-based lone pair (HOMO) and the vacant LUMO (Figure S15). Despite this stabilization, the Ti–C bond length is strikingly similar to that of **1**, which is likely due to the steric influence of the ^*t*^Bu residues on the rigidity of the pincer backbone. In the absence of ^*t*^Bu groups, using the truncated model (^H^POCOP)TiCl_3_, the same distortion from *C*_2_ symmetry leads to a substantial shortening of the Ti–C bond distance from 2.29 to 2.22 Å. This is not observed for (^H^PCP)TiCl_3_, for which the bond distance remains at 2.28 Å upon relaxation to *C*_1_ symmetry (Figure S5). Breaking the linkages between the bridges and phenyl ring causes the resulting complexes (HOP^*t*^Bu_2_)_2_Ti(Ph)Cl_3_ and (CH_3_P^*t*^Bu_2_)_2_Ti(Ph)Cl_3_ to relax to a distorted-octahedral structure, in which the mutually *trans*-located Cl/Cl and Cl/phenyl ligands distort toward the phosphorus ligands (Figure S6).^26^ A more detailed analysis using the Morokuma/Ziegler energy decomposition scheme provides further insight into the interactions between the ligands and metal fragments (see the Supporting Information). In conclusion, the meridional coordination mode of the pincer ligands restricts the conformational freedom of the complex, and the observed geometries are a result of a nuanced balance between steric and electronic factors.

**Scheme 2:**
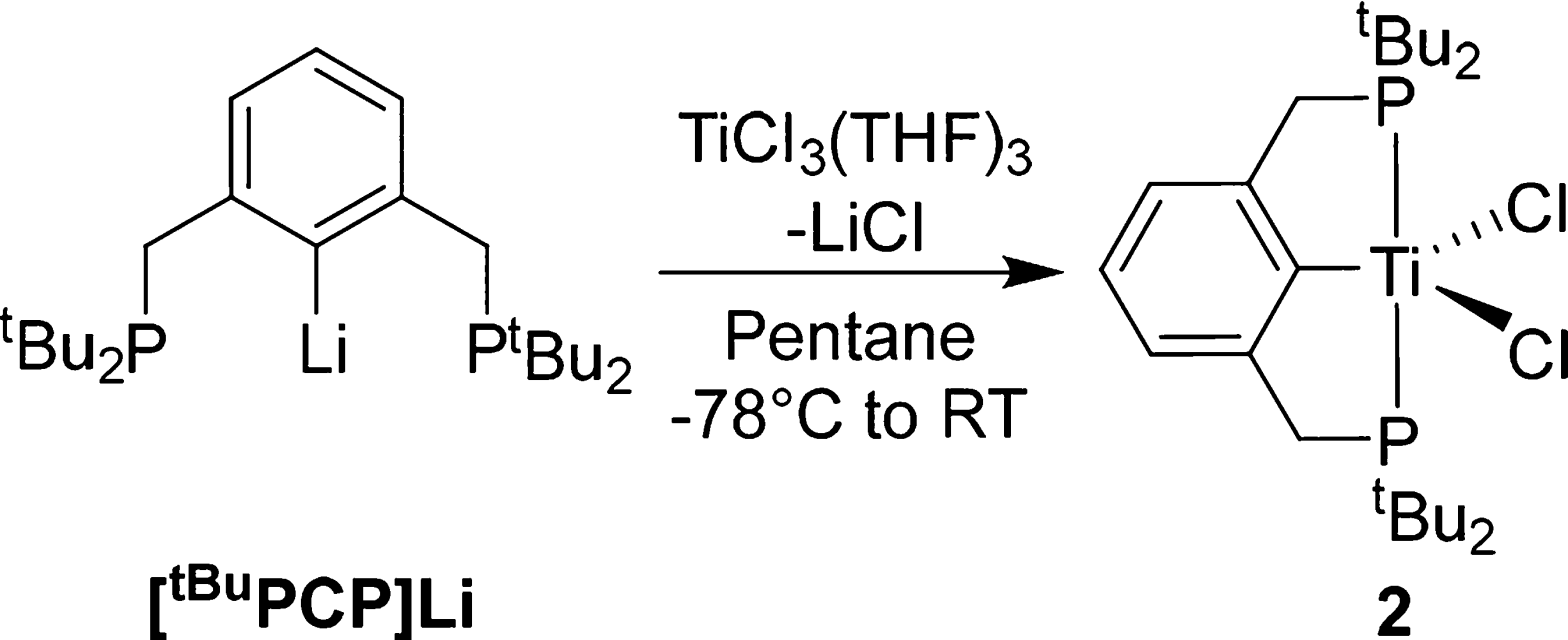
Synthesis of **2** An analogous synthesis in toluene was recently reported.

Given the ease of reduction of **1** to form **2**, we wanted to further investigate the synthesis and reactivity of this species as a “(PCP)Ti” synthon. Recently Hu et al. described the synthesis and crystal structure of (^tBu^PCP)TiCl_2_ (**2**) from the reaction of [^tBu^PCP]Li with TiCl_3_(THF)_3_ in toluene, achieving a 37% yield. Significantly improved yields can be achieved by doing this reaction in pentane and crystallizing from pentane at −40 °C (66% isolated yield, Scheme 2).

**Figure 2:**
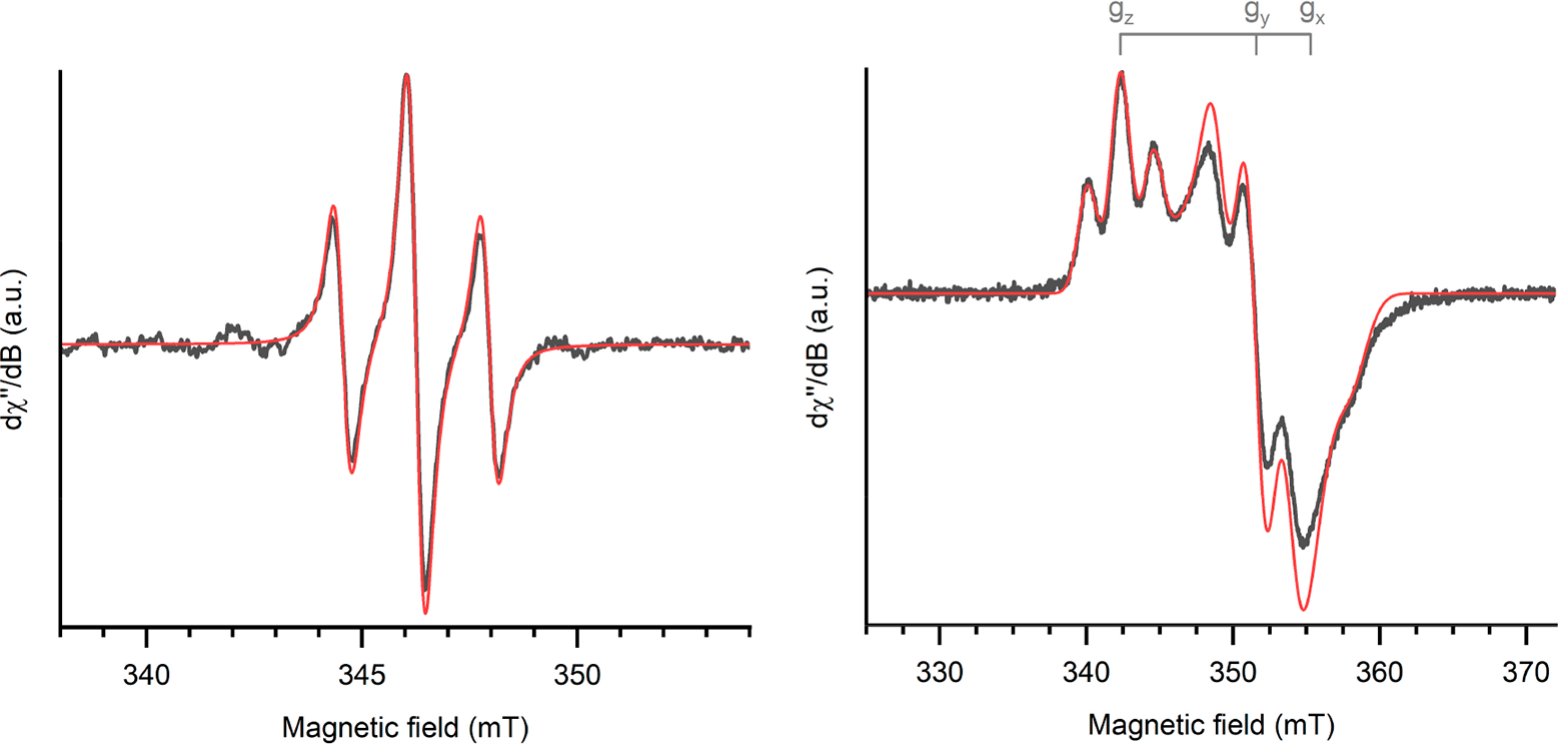
Continuous-wave X-band EPR spectra of **2** in toluene measured at 298 K (left; *g*_0_ = 1.9510, *a*_0_ = 1.71 mT) and 100 K (right, *g*_*x*_ = 1.9015 *g*_*y*_ = 1.9215, *g*_*z*_ = 1.9735, *A* = 2.6, 2.2, and 2.2 mT, respectively) measured in toluene (black, experimental; red, simulation).

^1^H NMR characterization of **2** is moderately uninformative, consisting primarily of broad humps. The ^31^P{^1^H} NMR spectrum displays no signals. The solution-phase magnetic moment of **2** (Evans method, C_6_D_6_) is 1.57 μ_B_—comparable to that of [2,5-(CH_2_P^t^Bu)_2_C_4_H_2_N]TiCl_2_.^14^ The room-temperature and frozen-matrix EPR spectra of **2** are shown in Figure 2.

The room-temperature spectrum is isotropic due to the fast tumbling in solution; freezing the sample allows the resolution of the rhombic symmetry of the electron Zeeman interaction. The single unpaired electron is predominantly located on the metal center (evidenced by *g*_0_ = 1.9510 at room temperature, significantly reduced from that of a carbon-centered radical and similar to that found for the analogous POCOP complex and the PNP-type complex [N(2-P(CHMe_2_)_2_-4-Me-C_6_H_3_)]TiCl_2_).^17,22^ There is, however, some spin density on the P centers, which gives rise to the triplet form of the room-temperature spectrum. Additionally, satellite lines are observed; these can be attributed to the hyperfine coupling with ^47^Ti (*I* = 5/2, 7.44% natural abundance) and ^49^Ti (*I* = 7/2, 5.41% natural abundance). Simulation of the room-temperature CW-EPR spectrum taking into account these interactions (Figure S3) allows us to estimate a coupling constant of 1.42 mT, equal for both isotopes because of their similar nuclear gyromagnetic ratios; this value is in substantial agreement with the previously reported value for Ti(III) chelates in aqueous solution.^46^

**Figure 3:**
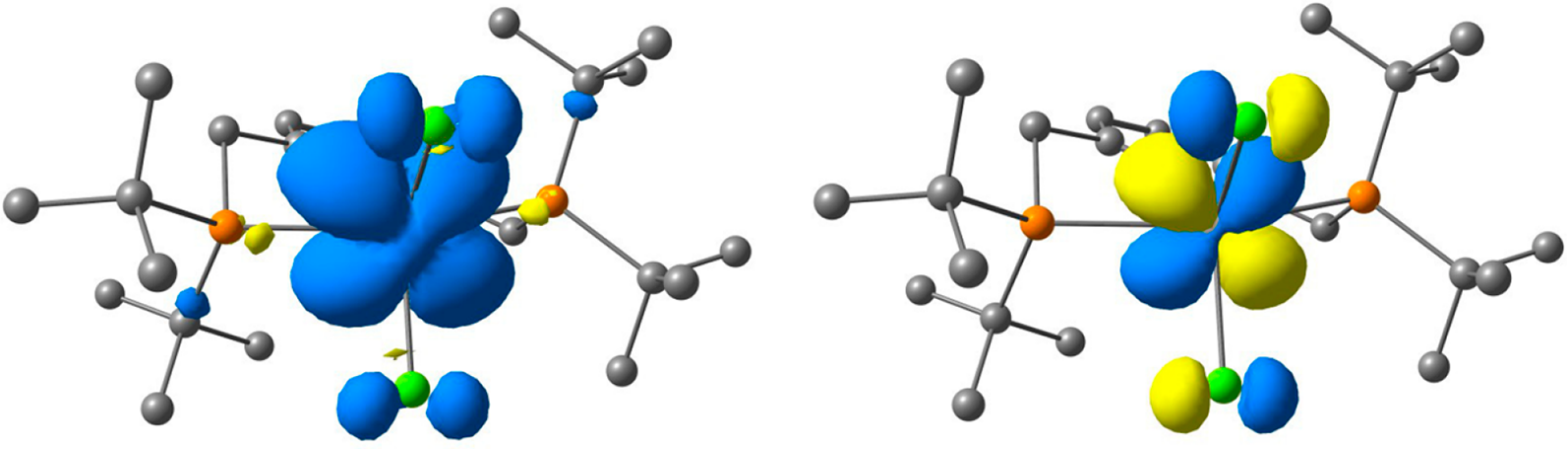
(left) Calculated spin density surface (isosurface 0.001 au) and (right) α-spin SOMO (isosurface 0.05 au) of **2** (B3LYP-D3(BJ)/def2-TZVPP). Hydrogen atoms omitted for clarity.

Upon freezing, the spectrum becomes rhombic, giving three overlapping triplets (Figure 2, right). The electronic structure of complex **2** was further verified using spin-unrestricted DFT calculations (Figure S8). The electronic ground state of **2** is a spin doublet with *S* = 1/2, with the unpaired electron residing in a largely nonbonding *d*_*xz*_ MO centered on Ti, with a small admixture of Cl 3*p* character (Figure 3).

**Scheme 3:**
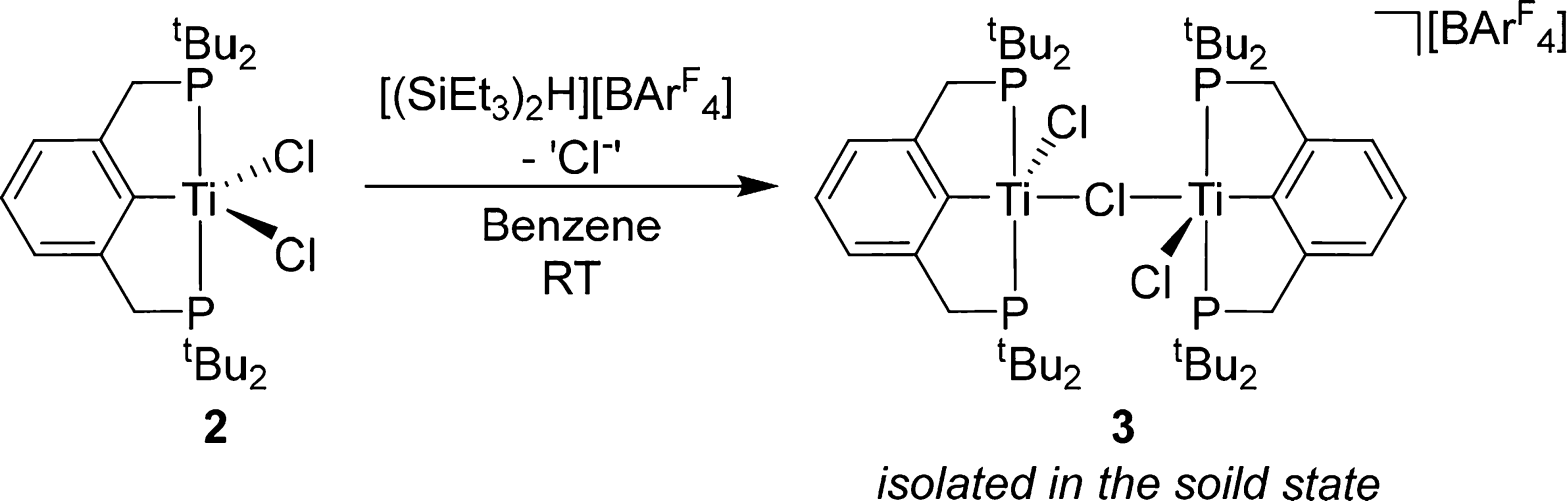
Synthesis of **3** by Halide Abstraction from **2** by [(SiEt_3_)_2_(μ-H)][BAr^F^_4_]

In an attempt to isolate a low-coordinate Ti(III) pincer complex, halide abstraction on **2** was attempted. In order to achieve this, [(SiEt_3_)_2_(μ-H)][BAr^F^_4_] (Ar^F^ = C_6_F_5_) was used—this has been used to great effect to abstract halides from particularly strong M–Cl bonds (e.g., lanthanides).^29−34^ Addition of [(SiEt_3_)_2_(μ-H)][BAr^F^_4_] to **2** in benzene resulted in the immediate formation of a brown oil, which could be extracted with fluorobenzene as a deep brown-red solution. A structural analysis found that only half an equivalent of chloride had been abstracted and a dimeric species could be isolated in the solid state, [((^tBu^PCP)TiCl)_2_(μ-Cl)][BAr^F^_4_] (**3**, Scheme 3). Even use of an excess of the halide abstraction agent did not alter the resultant product, exclusively forming the bridged dimer structure. **3** is insoluble in aliphatic and nonhalogenated aromatic hydrocarbon solvents.

**Figure 4:**
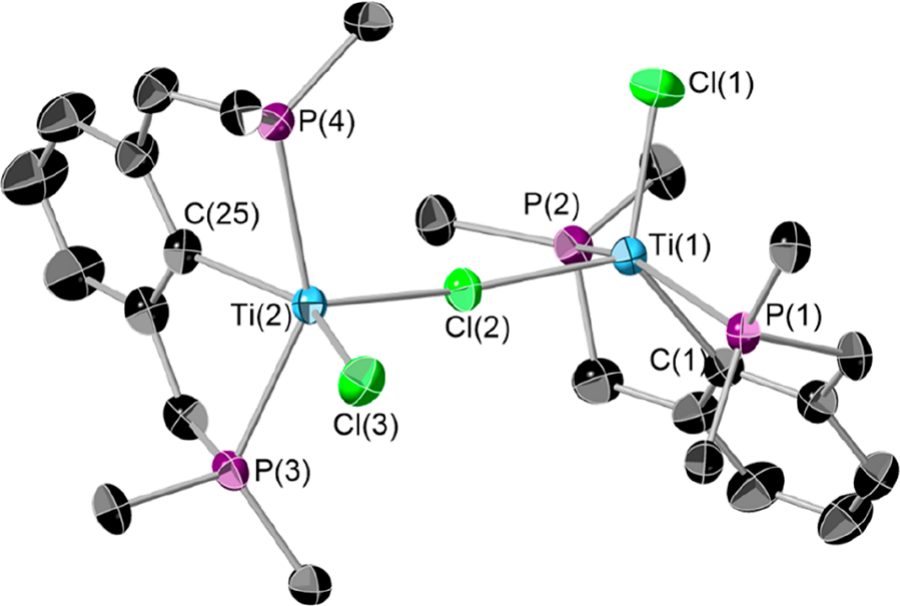
Single-crystal X-ray structure of **3**. The thermal ellipsoids are at 50% probability. The [BAr^F^_4_]^−^ anion, hydrogen atoms, and methyl groups on the *tert*-butyl substituents of the phosphines are omitted for clarity. Selected bond lengths (Å) and angles (deg): Ti(1)–Cl(1) 2.2591(7), Ti(1)–Cl(2) 2.4758(7), Ti(1)–P(1) 2.6357(7), Ti(1)–P(2) 2.6199(7), Ti(1)–C(1) 2.173(2), Ti(1)–Ti(2) 4.8972(6), Ti(2)–Cl(2) 2.4378(7), Ti(2)–Cl(3) 2.2719(7), Ti(2)–P(3) 2.5924(7), Ti(2)–P(4) 2.6374(7), Ti(2)–C(25) 2.187(2); Cl(1)–Ti(1)–C(1) 128.19(7), P(1)–Ti(1)–P(2) 151.03(2), Cl(1)–Ti(1)–Cl(2) 105.77(3), P(1)–Ti(1)–Cl(2) 95.56(2), P(2)–Ti(1)–Cl(2) 100.51(2), C(1)–Ti(1)–Cl(2) 126.02(7), Ti(1)–Cl(2)–Ti(2) 170.62(3), Cl(3)–Ti(2)–C(25) 147.76(7), P(3)–Ti(2)–P(4) 146.80(2), Cl(2)–Ti(2)–Cl(3) 104.82(3), Cl(2)–Ti(2)–P(3) 101.05(2), Cl(2)–Ti(2)–P(4) 106.10(2), Cl(2)–Ti(2)–C(25) 107.10(7).

Single crystals of **3** suitable for X-ray diffraction were grown by layering a fluorobenzene solution with hexane at room temperature. The resultant structure is shown in Figure 4.

The two titanium centers both adopt a square-based-pyramidal geometry, although one, Ti(1), is more distorted (τ = 0.067 and 0.016, Ti(1) and Ti(2), respectively). The PCP ligands are almost perpendicular to one another, demonstrated by the angle between the C(1)–Ti(1)–Cl(2) and C(25)–Ti(2)–Cl(2) planes: 79.58°. The central chloride is almost linear (Ti(1)–Cl(2)–Ti(2) = 170.623°). **3** is unique in that it is the only structurally characterized Ti dimer where the metal centers are only bridged by a single chlorine atom. Similar formulations have been invoked though not structurally characterized.^35^ Vertex-sharing Ti–F–Ti motifs exist, though these tend to be in fluoridotitanate ([Ti_*x*_F_*y*_]^*n*−^) anions, not in organometallic complexes.^36−40^ The individual bond lengths are similar to those observed in **2**.

Frustratingly, reliable EPR data of **3** could not be recorded. Any attempt at gathering such data resulted in multiple signals, giving evidence of more than one Ti(III) species existing in solution. It is likely that this is partially due to solvent interactions with the dimeric structure of **3**, breaking it apart; however, it could also be due to other side products in the reaction (which may explain the poor isolated yield of **3**).

The electronic structure of **3** was further investigated by DFT calculations. Optimized geometries of the complex in isoenergetic high-spin (*S* = 1) and broken-symmetry (*M*_s_ = 0) spin states reproduce the crystallographic structure well (Figure S9). The calculated bond parameters are similar for both, implying the presence of two independent fragments with local spin centers (*S*_loc_ = 1/2) on each Ti atom. The calculated Ti···Ti separation is somewhat shorter in the optimized geometries (∼4.65 Å) than in experiment (∼4.90 Å), which is attributed to the impact of the surrounding crystal matrix on the molecular structure. The resulting canonical frontier molecular orbitals and spin densities are consistent with two Ti(III) centers in which each *d*^1^-electron resides in an orbital of *d*_*yz*_ character (assuming a local Cartesian coordinate system), while the remaining d orbital manifold remains vacant (Figures S16 and S17).

**Scheme 4:**
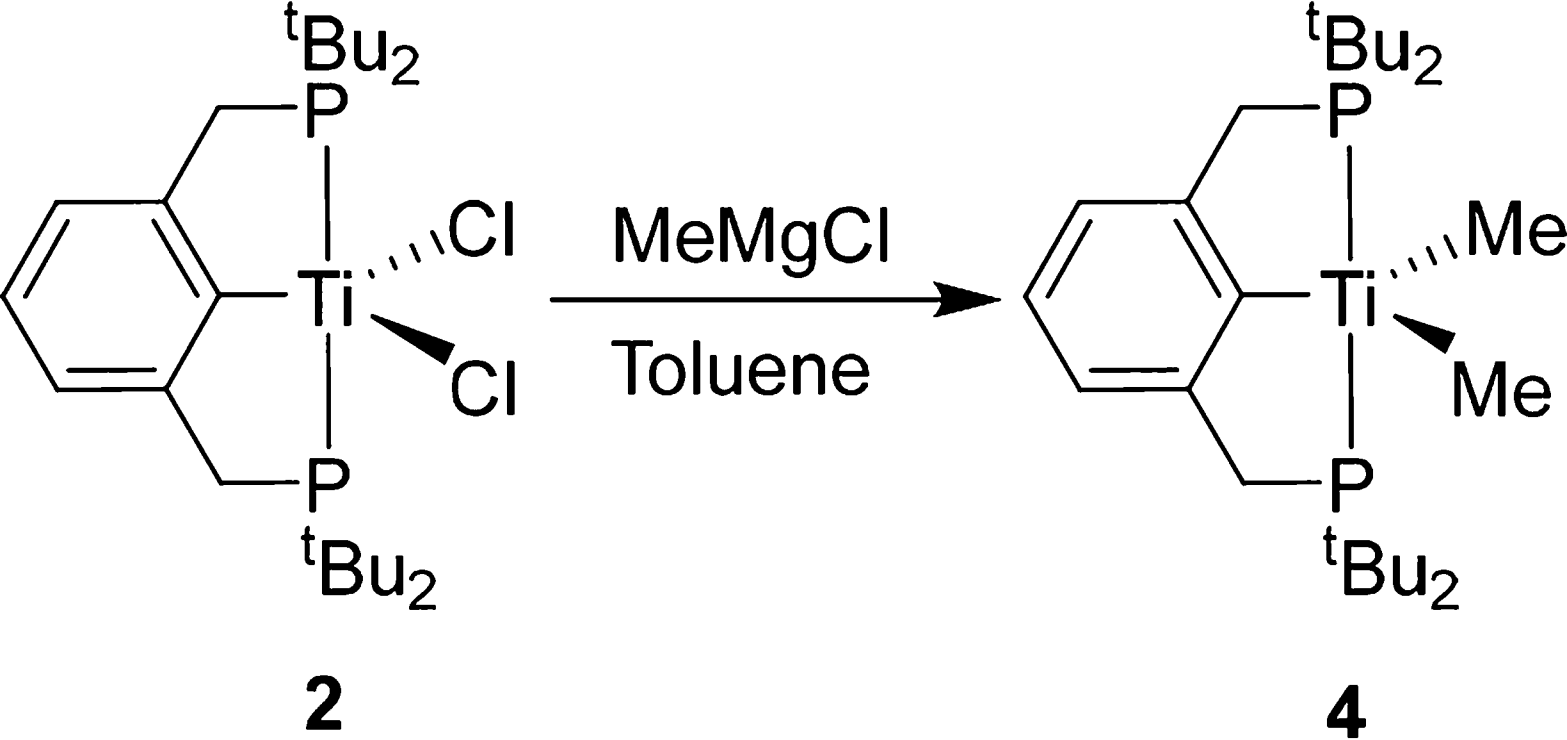
Synthesis of (^tBu^PCP)TiMe_2_ (**4**) from **2**

We also wanted to investigate the ability of **2** to act as a synthon for further “(PCP)Ti” metathesis chemistry. Reaction of **2** with MeMgCl in toluene resulted in a color change from blue to green, forming (^tBu^PCP)TiMe_2_ (**4**) (Scheme 4). **4** can be isolated in good yields (82%). When the reaction was attempted with 1 equiv of MeMgCl, **4** was still the only observed product (as well as unreacted **2**). The ^31^P{^1^H} NMR spectrum is featureless, and the ^1^H NMR spectrum consists of four broad humps centered at δ 24.04, 9.06, 2.35, and −1.33 ppm. A solution-phase magnetic moment of μ_eff_ = 1.60 μ_B_ was calculated (Evans method, C_6_D_6_), which is slightly lower than that of the closely related [N(2-P(CHMe_2_)_2_-4-Me-C_6_H_3_)]TiMe_2_ complex (1.82 μ_B_).^41^

**Figure 5:**
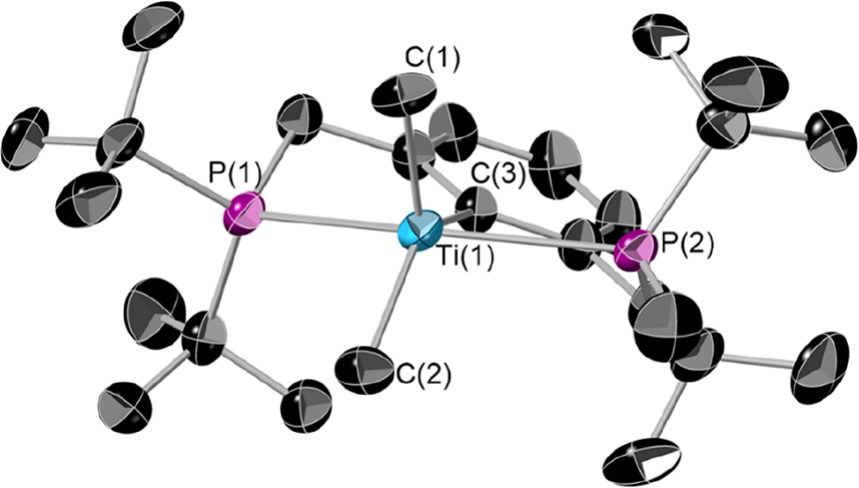
Single-crystal X-ray structure of **4**. The thermal ellipsoids are at 50% probability. Hydrogen atoms are omitted for clarity. Selected bond lengths (Å) and angles (deg): Ti(1)–C(1) 2.133(4), Ti(1)–C(2) 2.170(4), Ti(1)–C(3) 2.229(3), Ti(1)–P(1) 2.6355(11), Ti(1)–P(2) 2.6762(11), P(1)–Ti(1)–P(2) 148.69(4), P(1)–Ti(1)–C(1) 101.55(13) C(2)–Ti(1)–C(3) 132.15(15).

Single crystals of **4** suitable for X-ray diffraction can be grown by cooling a saturated pentane solution to −40 °C (Figure 5). Similarly to **2**, **4** adopts a distorted-square-pyramidal structure (τ = 0.28). It is interesting to note that the analogous POCOP complex adopts a near-perfect square-based-pyramidal structure. The bond lengths to the titanium center are in line with those observed for **2**, other “(PCP)Ti” complexes, and other Ti(III) methyl complexes.^17,42^

**Figure 6:**
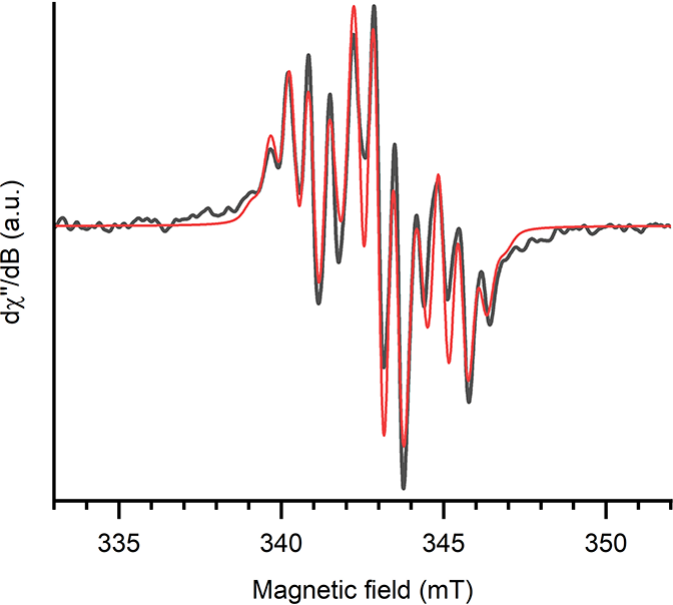
Continuous-wave X-band EPR spectrum of **4** (black, experimental; red, simulation) measured in toluene at 298 K.

The continuous wave X-band EPR spectrum, as well as the simulated spectrum, is shown in Figure 6. The signal is a triplet of septets, with *g*_0_ = 1.9685. The unpaired electron couples to the two ^31^P centers (*a*_0_ = 2.00 mT) and to the six methyl protons (*a*_0_ = 0.57 mT), resulting in the observed triplet of septets.

## Conclusions

The ^R^PCP ligand has been shown to bind to Ti(IV), and its coordination chemistry to Ti(III) has also been further developed. This work continues to extend the use of the progenitor pincer ligand to the group 4 metals. For Ti(III) complexes EPR has been shown to be an extremely useful diagnostic tool in their reactivity. For example, a halide can be abstracted from (^tBu^PCP)TiCl_2_ to give a chloride-bridged dimer in the solid state; however, EPR indicates that this species is broken apart by solvent interactions in the solution phase. The Ti(IV) complexes do not show the propensity to rearrange, unlike their POCOP cousins. We envisage these compounds to be useful starting points for further (PCP)Ti chemistry: in particular, their application in nonmetallocene polymerization catalysis and small-molecule activation. This study demonstrates that it is possible to alter the other ligands around the Ti center with the pincer acting as an innocent spectator, which will allow for fine-tuning of the metal’s steric and electronic profile.

## Experimental Section

Unless otherwise stated, all manipulations were carried out under an inert atmosphere (N_2_) using standard dual-manifold Schlenk techniques or employment of an MBraun Labmaster glovebox. Glassware was dried in an oven at 180 °C overnight before use. Anhydrous solvents (toluene, pentane, CH_2_Cl_2_) were obtained from a Grubbs type SPS system and stored over activated 3 Å molecular sieves (CH_2_Cl_2_) or potassium mirrors (toluene, pentane) under an inert atmosphere. THF and Et_2_O were dried by refluxing over Na/fluorenone and stored over activated 3 Å molecular sieves or a potassium mirror, respectively, while being kept under an inert atmosphere. Benzene was purchased as anhydrous and then transferred to an ampule over a potassium mirror and subsequently degassed by three freeze–pump–thaw cycles. Fluorobenzene was dried over CaH_2_, distilled onto 3 Å molecular sieves, and degassed by three further freeze–pump–thaw cycles. All other solvents, including deuterated solvents, were dried by being stored over activated 3 Å molecular sieves and subsequent degassing by three freeze–pump–thaw cycles.

Solution NMR data were collected on a Bruker 400 MHz spectrometer employing NMR tubes fitted with a J. Young style stopcock. Data were collected at room temperature. Chemical shifts (δ) are stated in ppm and referenced internally to residual solvent protio resonances (^1^H) or externally to 85% H_3_PO_4_ (^31^P) or LiCl (^7^Li). Coupling constants (*J*) and line widths are quoted in Hz. The data were processed using MestReNova.

X-band CW-EPR measurements were collected on a Magnettech ESR5000 spectrometer (Bruker) equipped with a TCH04 temperature controller. X-band EPR spectra were recorded using the following parameters: (^tBu^PCP)TiCl_2_ (**2**), 298 K, 100 mW microwave power, 0.1 mT field modulation amplitude at 100 kHz, 0.21 mT/s field sweep rate; (^tBu^PCP)TiCl_2_ (**2**), 100 K, 0.1 mW microwave power, 0.1 mT field modulation amplitude at 100 kHz, 0.21 mT/s field sweep rate; (^tBu^PCP)TiMe_2_ (**4**), room temperature, 10 mW microwave power, 0.2 mT field modulation amplitude at 100 kHz, 0.83 mT/s field sweep rate. All data analysis was carried out using EasySpin.^43^

Single-crystal X-ray diffraction data were collected as follows: a typical crystal was mounted on a MiTeGen Micromount using perfluoropolyether oil and cooled rapidly to 173 K in a stream of nitrogen gas using a cryostream unit. Data were collected with an Agilent Diffraction Xcalibur PX Ultra A or an Xcalibur 3 E diffractometer (Cu Kα radiation, λ = 1.54180 Å). Raw frame data were reduced using CrysAlisPro. The structures were solved using SuperFlip and refined using full-matrix least-squares refinement on all *F*^2^ data using the CRYSTALS program suite. In general, distances and angles were calculated using the full covariance matrix.

^tBu^PCP-Br, [(SiEt_3_)_2_(μ-H)][BAr^F^_4_], TiCl_4_(THF)_2_, and TiCl_3_(THF)_3_ were prepared by literature procedures.^18,30,44,45^ N.B.: TiCl_4_ is highly moisture reactive and can be dangerous if not handled correctly. ^n^BuLi and MeMgCl were purchased from commercial suppliers and titrated before use. Computational details can be found in the Supporting Information.

### Synthesis of [^tBu^PCP]Li

[^tBu^PCP]Li was prepared using a method adapted from previous reports.^18,21^^n^BuLi (1 mL, 2.5 M, 2.50 mmol, 1.25 equiv) was added to (^tBu^PCP)Br (989 mg, 2.09 mmol, 1.00 equiv) in pentane (25 mL) at −78 °C. The reaction mixture was warmed to room temperature and stirred for 2 days to afford a yellow solution with a colorless precipitate. The white solid was isolated by filtration, washed with pentane (3 × 10 mL), and then dried under vacuum to give [^tBu^PCP]Li (622 mg, 1.55 mmol, 81%) as a white powder. ^31^P{^1^H} NMR (400 MHz, THF): δ 26.99 (s). ^1^H NMR (400 MHz, C_6_D_6_/THF): δ 7.46 (br m, 2H, phenyl-H_m_), 6.89 (t, ^2^*J*_HH_ = 2.59 Hz, 1H, phenyl-H_p_), 3.36 (d, ^2^*J*_HP_ = 2.39 Hz, 4H, −CH_2_), 1.43 (d, ^3^*J*_HP_ = 9.94 Hz, 36H, −CH_3_). ^7^Li{^1^H} NMR (400 MHz, THF): δ 2.66 (s).

### Synthesis of (^tBu^PCP)TiCl_3_ (**1**)

To a suspension of TiCl_4_(THF)_2_ (291 mg, 0.874 mmol) in Et_2_O (25 mL) cooled to −78 °C was added dropwise a suspension of (^tBu^PCP)Li (355 mg, 0.874 mmol) in Et_2_O (25 mL). The mixture was kept cold for 1 h and then slowly warmed to room temperature and stirred overnight, affording a murky dark red-brown solution. The solution was filtered, the volatiles were removed under vacuum, and the resultant red-brown residue was extracted with pentane (3 × 10 mL). The deep red pentane solution was stored in a 4 °C refrigerator overnight and the supernatant decanted to afford deep red crystals of (^tBu^PCP)TiCl_3_. A crude yield of 23% could be achieved; however, the NMR data always show the presence of **2** in the sample. ^1^H NMR (C_6_D_6_): 6.90 (m, 1 H, Ar-H), 6.69 (d, ^2^*J*_HH_ = 5.7 Hz, 1 H, Ar-H), 3.37 (s, 4 H, PCH_2_), 1.28 (s, 36 H, CCH_3_). ^31^P{^1^H} NMR (C_6_D_6_): 91.78 (s). No accurate elemental analysis has been obtained due to persistent contamination with **2**.

### Synthesis of (^tBu^PCP)TiCl_2_ (**2**)

A suspension of (^tBu^PCP)Li (521 mg, 1.30 mmol, 1.00 equiv) in pentane (10 mL) was added dropwise to a suspension of TiCl_3_(THF)_3_ (530 mg, 1.43 mmol, 1.10 equiv) in pentane (5 mL) at −78 °C. Ten minutes after addition, the resulting mixture was warmed to room temperature and a change from light blue to a suspension of a black solid in a blue solution was observed. The mixture was left to react for 17 h, and then the blue solution was isolated by filtration and the solid washed with pentane (3 × 15 mL). The pentane was removed from the combined filtrates under vacuum to give a blue solid. This was redissolved in the minimum volume of pentane, filtered, and cooled to −40 °C to yield overnight blue needles of (^tBu^PCP)TiCl_2_ (440 mg, 0.86 mmol, 66%). ^1^H NMR (400 MHz, C_6_D_6_): δ 24.04 (br s, LW at fwhm = 304.72 Hz, 4H), 9.06 (br s, LW at fwhm = 26.19 Hz, 1H), 2.35 (br s, LW at fwhm = 113.82 Hz, 36H), −1.33 (br s, LW at fwhm = 59.43 Hz, 2H). No ^31^P NMR resonances were identified. Magnetic susceptibility (Evans method): μ_eff_ = 1.57 μ_B_ in C_6_D_6_ at 297 K. CW-EPR (9.4551 GHz, toluene, 298 K): , isotropic, *g*_0_ = 1.9510, *a*_0_ = 46.6 MHz (1.71 mT; coupling to two equivalent *I* = 1/2 nuclei), Voigt lineshape with LW = 0.26 mT at FWHM (Gaussian). IR (ATR, solid state): 2939, 2892, 2861, 1594, 1546, 1464, 1456, 1400, 1391, 1364, 1259, 1227, 1173, 1099, 1094, 1018, 1002, 956, 932, 887, 813, 810, 786, 746, 701, 612, 569, 514 cm^–1^. Elemental analysis found (calculated): C 56.60 (56.27); H 8.28 (8.46). No signals were observed in HRMS.

### Synthesis of [(^tBu^PCPTiCl)_2_(μ-Cl)][BAr^F^_4_] (**3**)

To (^tBu^PCP)TiCl_2_ (100 mg, 0.195 mmol) in benzene (10 mL) was added [(SiEt_3_)_2_(μ-H)][BAr^F^_4_] (178 mg, 0.215 mmol) in benzene (10 mL), resulting in the immediate formation of a suspension of a red-brown oil. The mixture was stirred for 4 h, and then the volatiles were removed *in vacuo*. The resulting crude brown-gold powder could be isolated; however this appears to contain multiple species. **3** may be obtained by recrystallizing this crude mixture by dissolving it in fluorobenzene (0.5 mL) and layering with hexane. After 2 days this afforded brown needle single crystals of [{(^tBu^PCP)TiCl}_2_(μ-Cl)][BAr^F^_4_] suitable for X-ray diffraction (recrystallized isolated yield 28 mg, 0.0168 mmol, 17%). No ^1^H or ^31^P NMR resonances were identified. Magnetic susceptibility (Evans method): μ_eff_ = 0.039 μ_B_ in CD_2_Cl_2_ at 295 K. IR (ATR, solid state): 2919, 2871, 1640, 1593, 1511, 1456, 1410, 1372, 1273, 1215, 975, 833, 809, 773, 767, 755, 724, 683, 661, 609, 601, 572, 476, 431, 414, 404 cm^–1^. A satisfactory elemental analysis could not be achieved due to the highly air-sensitive nature of the compound. TOF MS ES+: 1075.2733 ([{(^tBu^PCP)TiCl}_2_(μ-Cl)]; [*M* + H + CH_2_Cl_2_]^+^; calcd 1075.3482.

### Synthesis of (^tBu^PCP)TiMe_2_ (**4**)

MeMgCl (3.0 M in THF, 0.14 mL, 0.41 mmol, 2.05 equiv) was added dropwise to a solution of (^tBu^PCP)TiCl_2_ (100 mg, 0.20 mmol, 1.00 equiv) in toluene (10 mL) at room temperature. The solution turned from blue to green upon addition and was stirred for 24 h. After 1,4-dioxane (0.5 mL, 5.87 mmol) was added, the reaction mixture was stirred for 30 min and filtered and then the volatiles were removed *in vacuo* to give a white solid and a green residue. Hexane (5 mL) was added to the solids, and the solution was filtered; then the undissolved white solid was washed with hexane (3 × 5 mL). The hexane was removed from the combined filtrates under vacuum to afford green solid (^tBu^PCP)TiMe_2_ (77.2 mg, 0.16 mmol, 82%). Single crystals suitable for X-ray diffraction could be grown by slow evaporation of a concentrated pentane solution over 7 days. ^1^H NMR (400 MHz, C_6_D_6_): δ 32.53 (br s, LW at fwhm = 240.34 Hz, 4H), 8.45 (br s, LW at fwhm = 13.37 Hz, 1H), 3.34 (br s, LW at fwhm = 73.54 Hz, 36H), 0.32 (br s, LW at fwhm = 24.60 Hz, 2H). No ^31^P resonances were identified. Magnetic susceptibility (Evans method): μ_eff_ = 1.60 μ_B_ in C_6_D_6_ at 295 K. CW-EPR (9.4502 GHz, 0.01 M in toluene, rt): isotropic, *g*_0_ = 1.9685, *a*_0__,1_ = 55.2 MHz G (2.00 mT; coupling to two equivalent *I* = 1/2 nuclei), *a*_0,2_ = 15.7 MHz (0.57 mT; coupling to six equivalent *I* = 1/2 nuclei), Voigt lineshape with LW = 0.46 mT at FWHM (Gaussian) + 0.30 mT at FWHM (Lorentzian). IR (ATR, solid state): 3033, 2978, 2943, 2891, 2864, 1547, 1461, 1430, 1388, 1365, 1310, 1227, 1178, 1091, 1008, 983, 935, 891, 839, 811, 783, 739, 704, 610, 571 cm^–1^. Elemental analysis found (calculated): C 66.54 (66.20); H 10.50 (10.48). TOF MS ES+: 472.4046 ([C_26_H_50_P_2_Ti]^+^; [*M* + H]^+^; calcd 472.2867).

## Supporting Information Available

Additional characterization data, crystallographic data, and computational details (PDF)
Computational coordinate data (XYZ)

## Supplementary Material

om2c00662_si_001.pdf

om2c00662_si_002.xyz
