# Supplementary material for: PCP Pincer Complexes of Titanium in the +3 and +4 Oxidation States
Source: Organometallics. 2023 Mar 13;42(12):1278–85. doi: 10.1021/acs.organomet.2c00662 (PMC10302872; doi:10.1021/acs.organomet.2c00662)
Supplement: Supplementary file 1 — om2c00662_si_001.pdf [file om2c00662_si_001.pdf]

Supplementary Information for “PCP pincer complexes of Titanium in the +3 and +4 oxidation state”

Benedek Stadler[‡a], Hilary H. Y. Meng[‡a], Sara Belazregue[a], Leah Webster[a], Alberto Collauto[a], Keelan M. Byrne[b], Tobias Krämer[b], F. Mark Chadwick[a]*

[a]Molecular Sciences Research Hub, Department of Chemistry, Imperial College London, 82 Wood Lane, London, W12 0BZ, United Kingdom

[b]Department of Chemistry, Maynooth University, Maynooth, Co. Kildare, Ireland.

[‡]These Authors Contributed Equally

Email: m.chadwick@imperial.ac.uk

1. Further Spectra

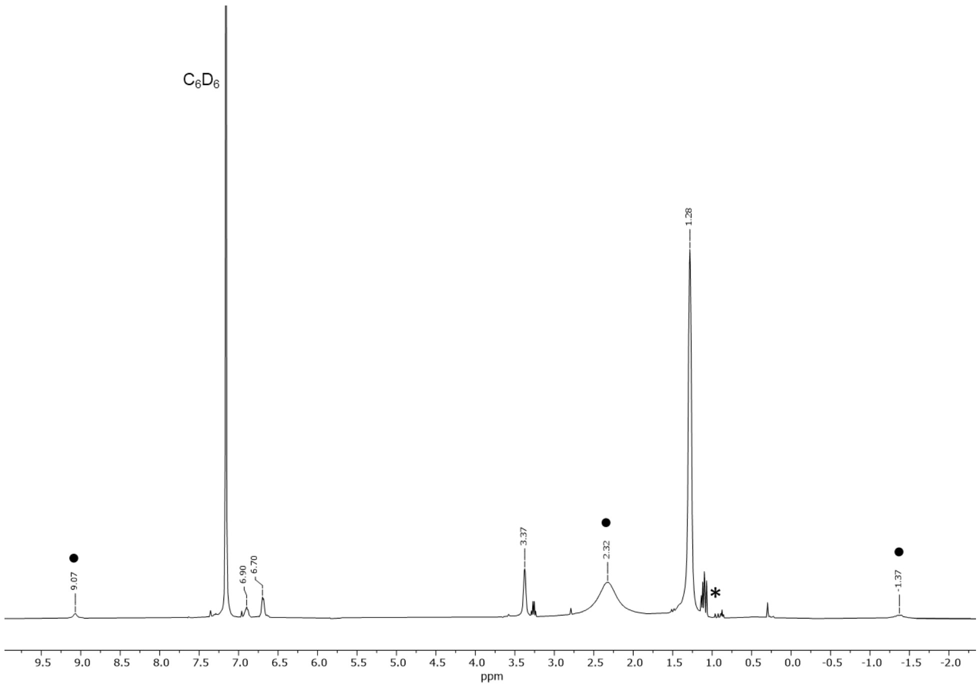

Figure S1: The ^{1}H NMR spectrum of **1**. Impurities are identified as 2 (•) and residual pentane (*).

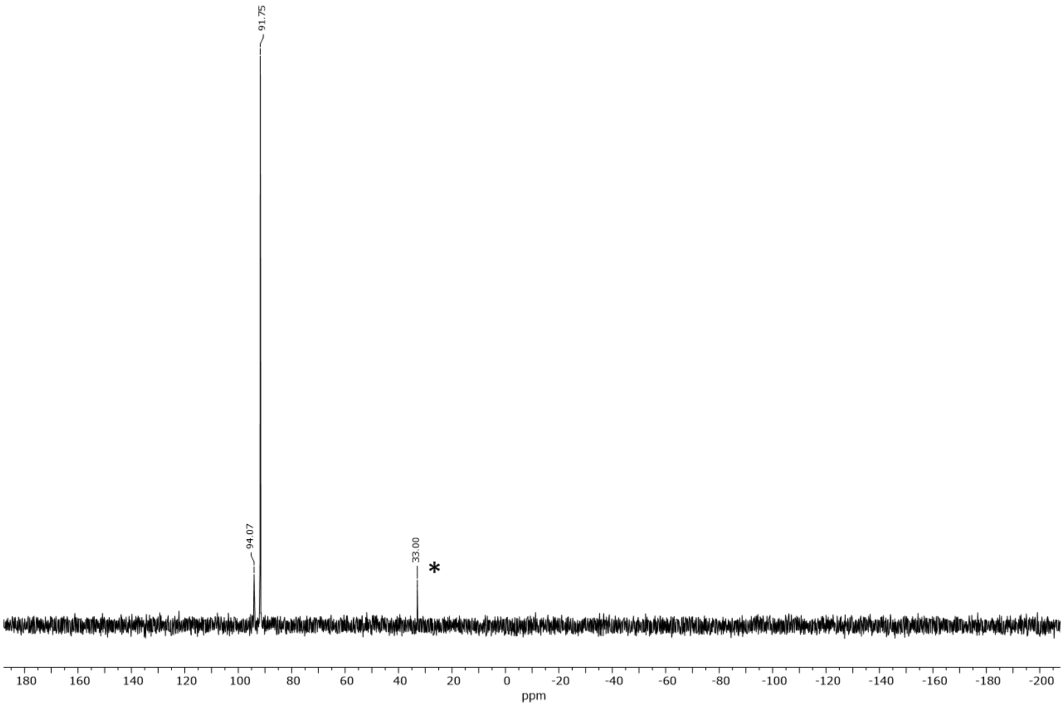

Figure S2: $^{31}P\{^{1}H\}$ NMR spectrum of **1**. Impurity marked * is due to protonated ligand. Impurity at 94 ppm is due to residual Li-PCP.

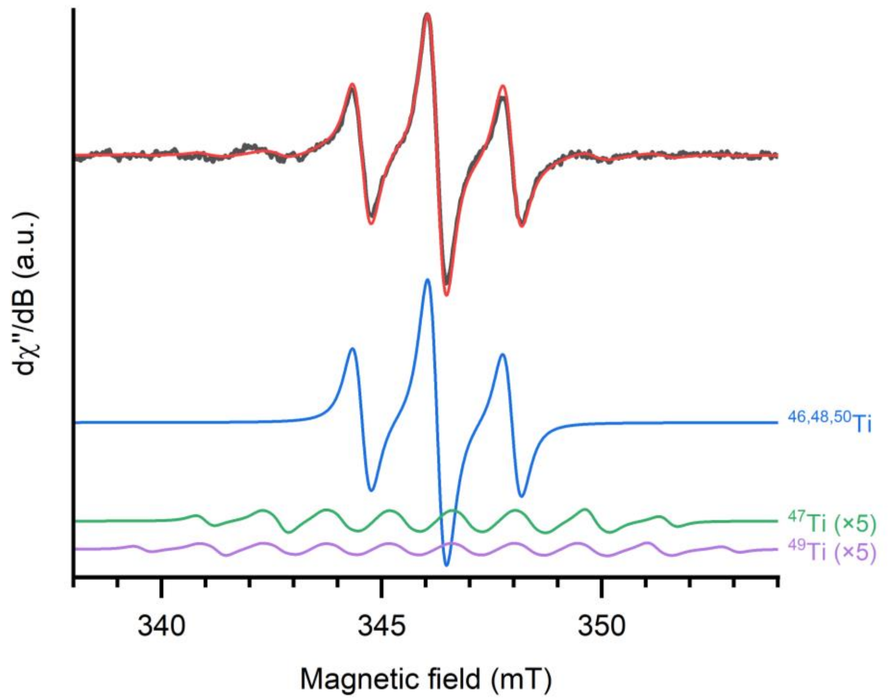

Figure S3: Simulation of the continuous-wave X-band EPR spectrum of **2** including the NMR-active isotopes of Ti (^{47}Ti: 7.44% natural abundance, I = 5/2; ^{49}Ti: 5.41% natural abundance, I = 7/2). Simulations parameters: g_0 = 1.9510, a_0(^{31}P) = 1.71 mT, a_0(Ti) = 1.42 mT. Black = experimental, red = simulation. For the sake of improved visualization, the components originating from the ^{47}Ti isotope (green) and ^{49}Ti isotope (purple) have been magnified by a factor of 5.

2. Further Crystallographic Details

Table S1. Experimental Crystal Data Details

	**(1)**	**(3)**	**(4)**
**Crystal data**			
Chemical formula	$C_{24}H_{43}Cl_3P_2Ti$	$C_{48}H_{86}Cl_3P_4Ti_2\cdot C_{24}BF_{20}$	$C_{26}H_{49}P_2Ti$
M_r	547.81	1668.30	471.52
Crystal system, space group	Monoclinic, $P2_1/c$	Triclinic, *P* -1	Monoclinic, $P2_1/n$
a, *b*, *c* (Å)	13.4035 (3),<br>15.2243 (3),<br>27.8928 (5)	14.2838 (4),<br>14.9654 (5),<br>19.8832 (7)	12.8480 (3), 16.5335 (5),<br>40.4982 (13)
α, β, γ (°)	90,<br>90.444 (2),<br>90	107.653 (3),<br>96.398 (2),<br>101.994 (3)	90,<br>94.278 (3),<br>90
V ($Å^3$)	5691.6 (2)	3891.7 (2)	8578.8 (4)
Z	8	2	12
m (mm^{-1})	6.26	4.24	3.64
Crystal size (mm)	0.20 × 0.20 × 0.10	0.40 × 0.20 × 0.05	0.40 × 0.40 × 0.10
**Data collection**			
T_{min}, T_{max}	0.37, 0.53	0.65, 0.81	0.26, 0.69
No. of measured, independent and observed [*I* > 2.0s(*I*)] reflections	20678,<br>10954,<br>9510	25131,<br>15059,<br>12432	27884,<br>16437,<br>13123
R_{int}	0.035	0.039	0.042
$(\sin q/l)_{max}$ ($Å^{-1}$)	0.621	0.622	0.623
**Refinement**			
$R[F^2 > 2s(F^2)]$,<br>$wR(F^2)$,<br>*S*	0.099,<br>0.287,<br>1.02	0.039,<br>0.106,<br>1.00	0.057,<br>0.170,<br>0.99
No. of reflections	10954	15053	16437
No. of parameters	541	919	784
$D\rho_{max}$, $D\rho_{min}$ (e $Å^{-3}$)	1.87, -0.93	0.49, -0.49	0.85, -0.88

3. Computational Results

3.1 Computational Methods

All electronic structure calculations were carried out using the Gaussian 16 (revision B.01) and the Amsterdam Density Functional 2022.102 (ADF) program packages.[1,2] Unconstrained optimizations of ground-state geometries and subsequent analytical frequency calculations were carried out at DFT level using the BP86 GGA exchange-correlation functional[3,4] in conjunction with Ahlrich's def2-TZVP basis set on Ti, P and Cl atoms and def2-SVP on C and H atoms.[5,6] Effects due to van-der-Waals interactions were accounted for by inclusion of Grimme's atom-pairwise dispersion correction including Becke-Johnson damping (D3BJ).[7,8] An ultrafine integration grid, corresponding to a pruned grid of 99 radial shells and 590 angular points per shell, was used for all calculations. All stationary points were confirmed to be minima by the absence of any imaginary mode in their vibrational spectra. Single-point calculations for accurate energies and electronic structure analysis were performed at the B3LYP-D3BJ/def2-TZVPP[9] level of theory utilising the previously optimised geometries. ETS-NOCV calculations were carried out using the ADF program package at the BP86-D4/TZP level of theory.[10-12]

3.2 Optimised Geometries

Table S2: Comparison of selected calculated and experimental bond parameters for complexes **1** and (^{tBu}POCOP)TiCl$_3$ (BP86-D3(BJ)/def2-TZVP/def2-SVP).

**1** (C_1)	**Exp.**	**DFT**	
		(C_1) S = 0	(C_2) S = 0
Ti(1)–Cl(1)	2.268(2)	2.289	2.300
Ti(1)–Cl(2)	2.318(2)	2.309	2.303
Ti(1)–Cl(3)	2.304(2)	2.307	2.300
Ti(1)–P(1)	2.679(2)	2.620	2.637
Ti(1)–P(2)	2.675(2)	2.671	2.637
Ti(1)–C(1)	2.204(7)	2.212	2.215
Cl(1)–Ti(1)–Cl(2)	92.16(10)	93.97	92.73
Cl(1)–Ti(1)–P(1)	91.17(8)	89.89	87.80
Cl(1)–Ti(1)–P(2)	87.64(8)	87.36	90.78
Cl(1)–Ti(1)–C(1)	89.90(2)	90.38	87.27
Cl(3)–Ti(1)–Cl(2)	90.02(10)	90.73	92.73
Cl(3)–Ti(1)–P(1)	87.95(8)	95.34	90.78
Cl(3)–Ti(1)–P(2)	92.10(8)	85.83	87.80
Cl(3)–Ti(1)–C(1)	88.0(2)	86.67	87.27
P(1)–Ti(1)–P(2)	149.77	149.72	149.79
Cl(2)–Ti(1)–C(1)	173.60(?)	162.13	180.00
Cl(1)–Ti(1)–Cl(3)	177.68	173.07	174.54
**(^{tBu}POCOP)TiCl$_3$**		(C_1) S = 0	(C_2) S = 0
Ti(1)–Cl(1)	2.264(11)	2.267	2.285
Ti(1)–Cl(2)	2.383(10)	2.356	2.288
Ti(1)–Cl(3)	2.301(10)	2.309	2.285
Ti(1)–P(1)	2.613(11)	2.612	2.644
Ti(1)–P(2)	2.633(11)	2.580	2.644
Ti(1)–C(1)	2.208(11)	2.220	2.217
Cl(1)–Ti(1)–Cl(2)	–	88.34	92.50
Cl(1)–Ti(1)–P(1)	–	84.69	88.19
Cl(1)–Ti(1)–P(2)	–	113.63	90.23
Cl(1)–Ti(1)–C(1)	–	87.08	87.50
Cl(3)–Ti(1)–Cl(2)	–	86.09	92.50
Cl(3)–Ti(1)–P(1)	–	79.45	90.23
Cl(3)–Ti(1)–P(2)	–	99.65	88.19
Cl(3)–Ti(1)–C(1)	–	116.05	87.50
P(1)–Ti(1)–P(2)	–	136.41	143.00
Cl(2)–Ti(1)–C(1)	–	145.64	180.00
Cl(1)–Ti(1)–Cl(3)	143.16(4)	144.36	175.01

**2**		S = 1/2	
Ti(1)–Cl(1)		2.296	
Ti(1)–Cl(2)		2.296	
Ti(1)–C(1)		2.231	
Ti(1)–P(1)		2.547	
Ti(1)–P(2)		2.547	
P(1)–Ti(1)–P(2)		148.49	
P(1)–Ti(1)–Cl(1)		103.36	
Cl(2)–Ti(1)–C(1)		126.22	
**3**		*S* = 1	M_S = 0
Ti(1)–Cl(1)	2.2591(7)	2.271	2.269
Ti(1)–Cl(2)	2.4758(7)	2.326	2.343
Ti(1)–P(1)	2.6357(7)	2.550	2.547
Ti(1)–P(2)	2.6199(7)	2.594	2.593
Ti(1)–C(1)	2.1730(2)	2.197	2.193
Ti(1)···Ti(2)	4.8972(6)	4.614	4.651
Ti(2)–Cl(2)	2.4378(7)	2.326	2.343
Ti(2)–Cl(3)	2.2719(7)	2.271	2.269
Ti(2)–P(3)	2.5924(7)	2.594	2.593
Ti(2)–P(4)	2.6374(7)	2.550	2.547
Ti(2)–C(25)	2.1870(2)	2.197	2.193
Cl(1)–Ti(1)–C(1)	128.19(7)	148.68	148.71
P(1)–Ti(1)–P(2)	151.03(2)	149.49	150.17
Cl(1)–Ti(1)–Cl(2)	105.77(3)	113.10	112.74
P(1)–Ti(1)–Cl(2)	95.56(2)	96.19	96.17
P(2)–Ti(1)–Cl(2)	100.51(2)	102.99	102.30
C(1)–Ti(1)–Cl(2)	126.02(7)	97.34	97.66
Ti(1)–Cl(2)–Ti(2)	170.62(3)	165.50	165.99
Cl(3)–Ti(2)–C(25)	147.76(7)	148.65	148.71
P(3)–Ti(2)–P(4)	146.80(2)	149.48	150.17
Cl(2)–Ti(2)–Cl(3)	104.82(3)	113.13	112.73
Cl(2)–Ti(2)–P(3)	101.05(2)	102.98	102.30
Cl(2)–Ti(2)–P(4)	106.10(2)	96.21	96.17
Cl(2)–Ti(2)–C(25)	107.10(7)	97.34	97.67
ρ(Ti1)<br>ρ(Ti2)		1.11<br>1.11	–0.93<br>+0.93
**4**		*S* = 1/2	
Ti(1)–C(1)	2.133	2.139	
Ti(1)–C(2)	2.170	2.139	
Ti(1)–C(3)	2.229	2.267	
Ti(1)–P(1)	2.635	2.567	
Ti(1)–P(2)	2.676	2.567	
P(1)–Ti(1)–P(2)	148.695	147.03	
P(1)–Ti(1)–C(1)	???	104.74	
C(2)–Ti(1)–C(3)	132.151	128.42	

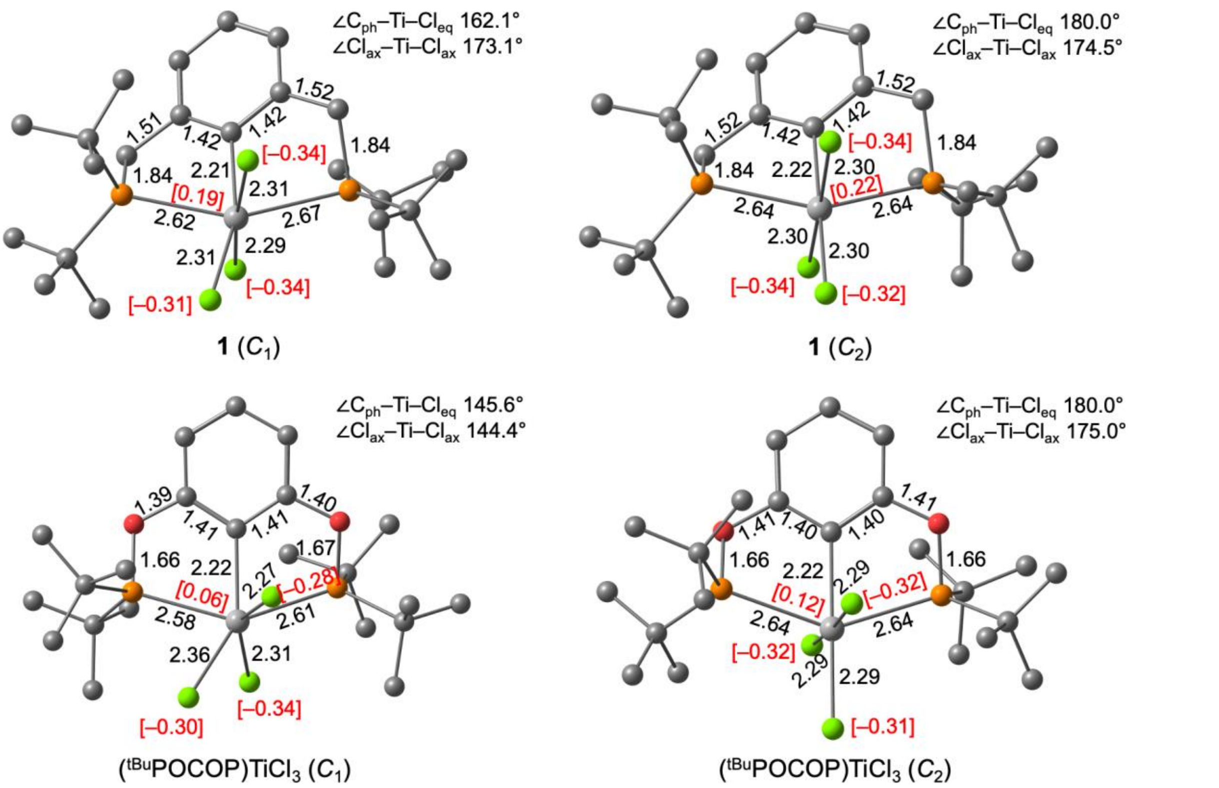

Figure S4: Optimised geometries (BP86-D3/def2-TZVP/def2-SVP) of **1** and (^{tBu}POCOP)TiCl$_3$ (bond distances in Å, Mulliken charges shown in red).

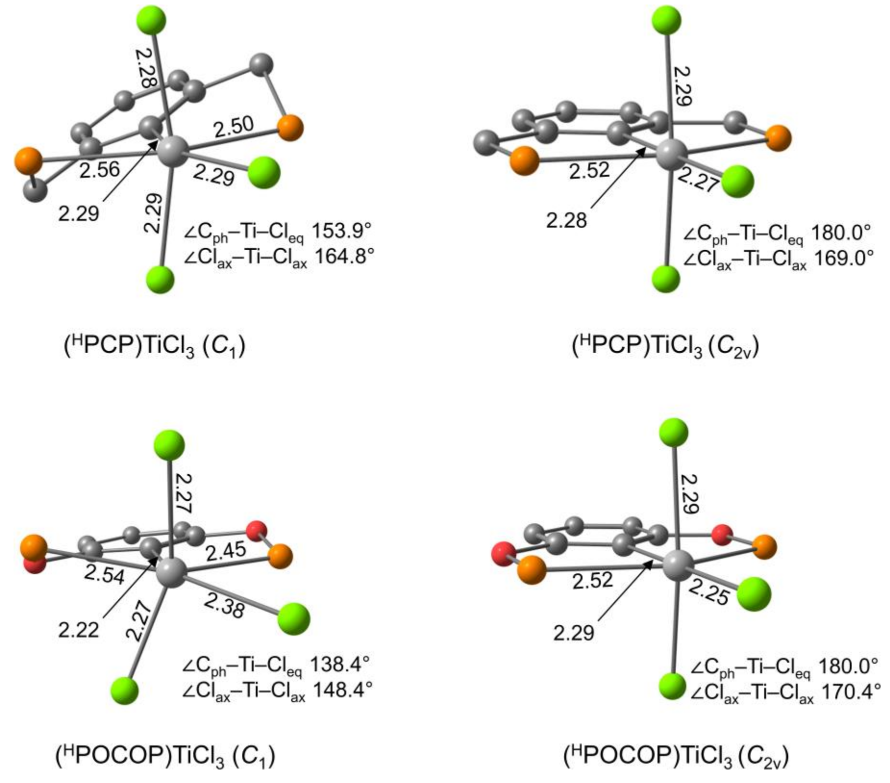

Figure S5: Optimised geometries (BP86-D3/def2-TZVP/def2-SVP) of (^{H}PCP)TiCl$_3$ and (^{H}POCOP)TiCl$_3$ (bond distances in Å).

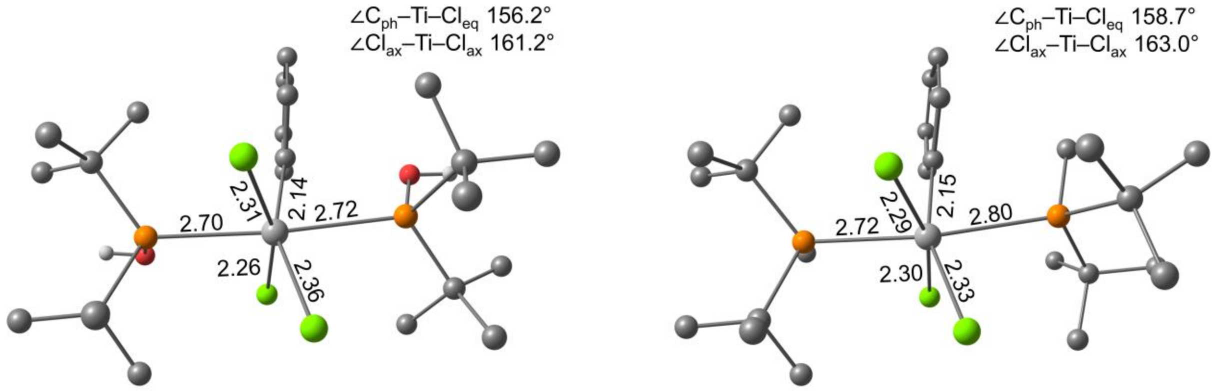

$(POH(^tBu)_2)_2Ti(Ph)Cl_3$ $(PCH_3(^tBu)_2)_2Ti(Ph)Cl_3$

Figure S6: Optimised geometries (BP86-D3/def2-TZVP/def2-SVP) of model complexes $(POH(^tBu)_2)_2Ti(Ph)Cl_3$ and $(PCH_3(^tBu)_2)_2Ti(Ph)Cl_3$ in which the bond between the bridging groups and phenyl have been broken and capped with H atoms (bond distances in Å).

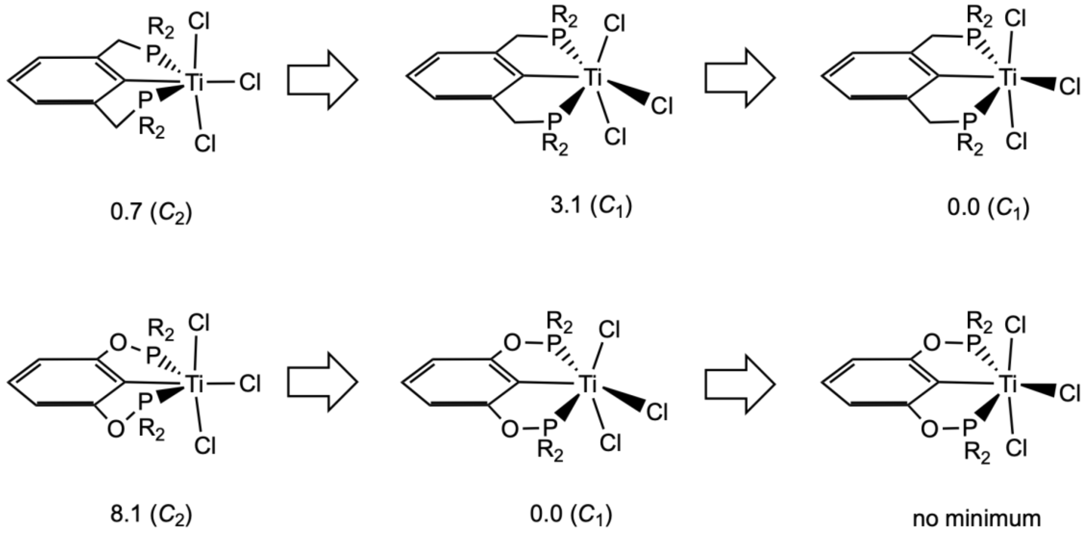

Figure S7: Relative energies (kcal mol^{-1}) of minima of **1** and (^{tBu}POCOP)$TiCl_3$ (BP86-D3/def2-TZVP/def2-SVP).

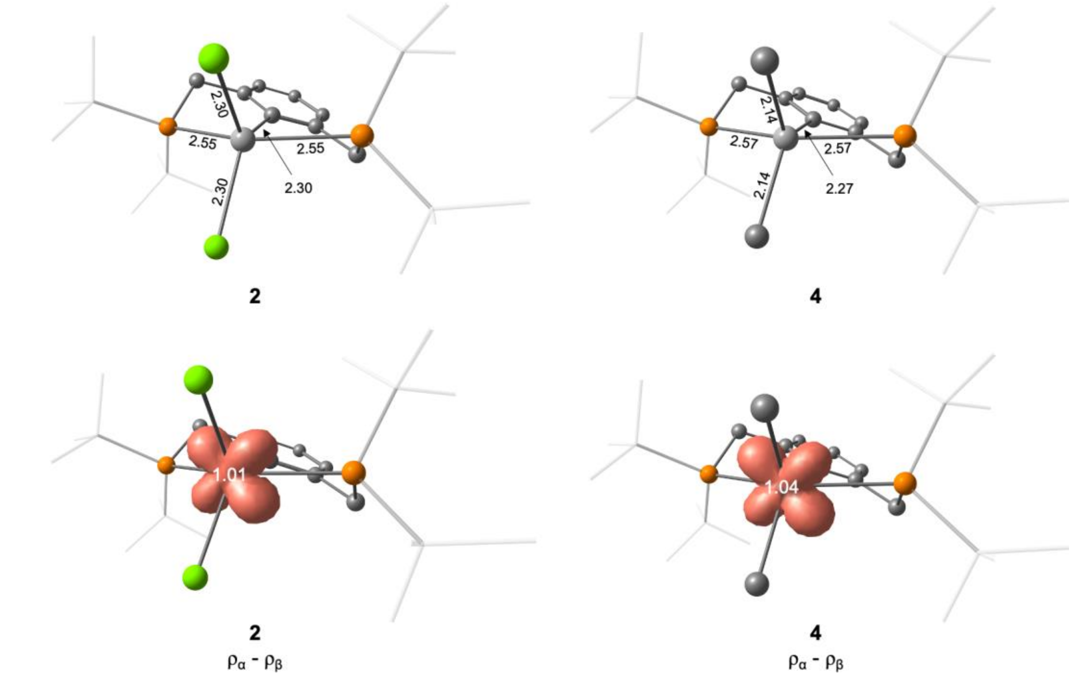

Figure S8: Optimised geometries (BP86-D3/def2-TZVP/def2-SVP) of complexes **2** and **4** (bond distances in Å), along with spin density plots (isovalue 0.02 au).

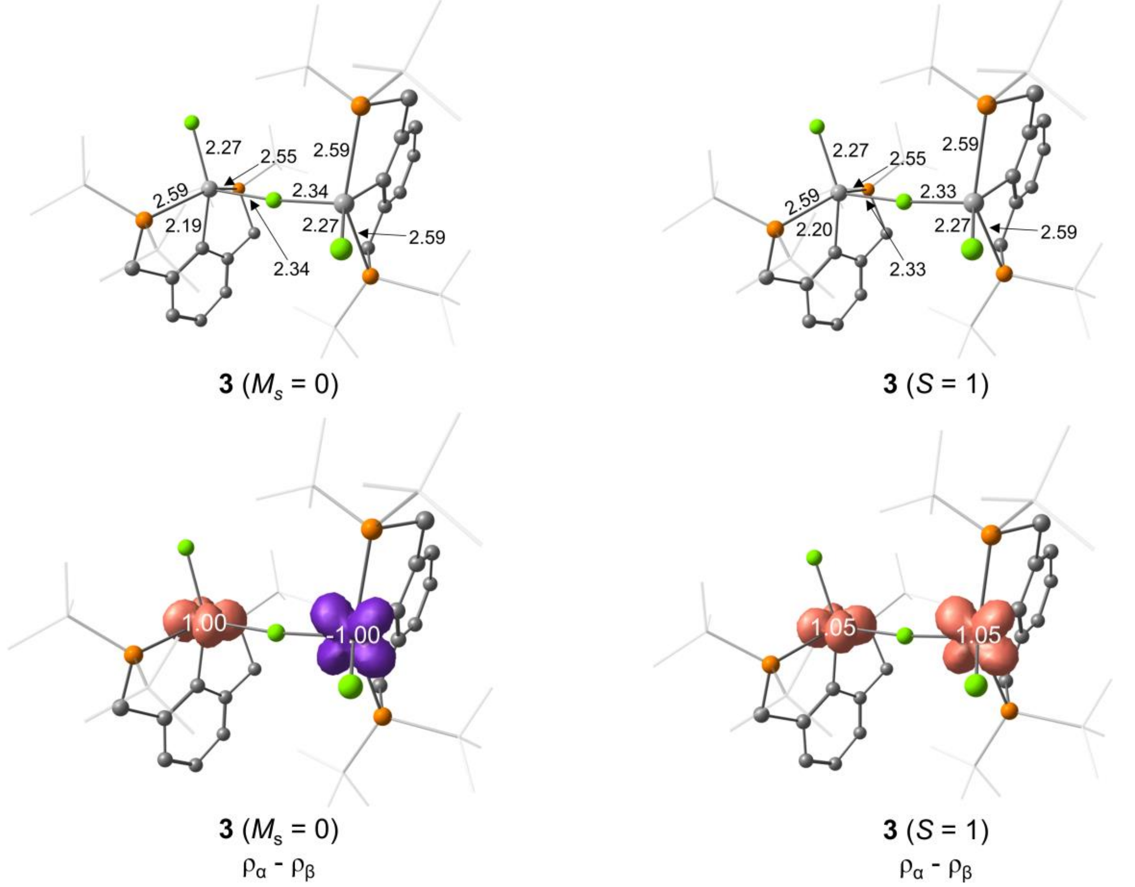

Figure S9: Optimised geometries (BP86-D3/def2-TZVP/def2-SVP) of complexes **3** in their triplet (S = 1) and broken-symmetry singlet states (M_s = 0). Selected bond distances are given in Å, along with spin density plots (isovalue 0.02 au).

3.3 Results of the Energy Decomposition Analysis

The interactions between the ligand and $TiCl_3$ fragments was analysed using Energy Decomposition Analysis (EDA) and the Extended Transition State/Natural Orbitals for Chemical Valence method (ETS-NOCV). To this end each of the complexes was partitioned into {$^{tBu}POCOP^{-}$} / {$^{tBu}PCP^{-}$} and {$TiCl_3^{+}$} fragments. Within the EDA scheme the bond energy between these fragments is expressed in terms of the preparation energy (strain or deformation energy) energy and the interaction energy:

$$\Delta E_{bond} = \Delta E_{prep} + \Delta E_{int}$$

The preparation energy ΔE_{prep} corresponds to the energy that is required to deform the fragments from their equilibrium structures to the geometry they have in the complex. The interactions energy ΔE_{int} is associated with the energy change when the prepared fragments are then combined to form the complex. A quantitative energy decomposition analysis (EDA) divides this interaction energy into several components: electrostatic interaction, Pauli repulsion, orbital interactions and a dispersion term:

$$\Delta E_{int} = \Delta E_{elstat} + \Delta E_{Pauliv} + \Delta E_{orb} + \Delta E_{disp}$$

The electrostatic interaction, usually attractive, is the energy between the unperturbed charge distributions of the prepared fragments. The Pauli repulsion is responsible for steric repulsion, it consists of the destabilizing interactions between occupied orbitals of the fragments. The orbital interaction accounts for charge transfer and polarization. The ETS-NOCV method provides an alternative charge / energy decomposition scheme that can be used to partition the deformation density into different orbital components. The (energy) contribution of each channel (σ, π etc.) to the total bond energy is evaluated for each specific orbital interactions between the fragments. Table S2 summarises the results of the EDA analysis of different symmetries of complexes **1** and (^{tBu}POCOP)$TiCl_3$.

Table S3: Energy decomposition analysis of **1** and (^{tBu}POCOP)$TiCl_3$ (BP86-D4/TZP) of in both C_1 and C_2 symmetric forms. $^{a}\Delta E_{bond}$ is defined as the sum of the preparation energy (ΔE_{prep}) and the interaction energy (ΔE_{int}. All energies stated are in units of kcal mol^{-1}.

	$\Delta E_{bond}{}^{a}$	ΔE_{prep}	ΔE_{int}	ΔE_{elstat}	ΔE_{Pauli}	ΔE_{orb}	ΔE_{disp}
**1** (C_1)	–250.30	59.49	–309.79	–290.65	233.59	–227.02	–25.71
**1** *(C_1, distorted)*	–248.47	67.35	–315.82	–289.38	237.05	–237.08	–25.23
**1** (C_2)	–249.87	59.18	–309.05	–288.46	228.32	–223.35	–25.56
(^{tBu}POCOP)$TiCl_3$ (C_1)	–239.24	60.46	–299.70	–277.94	239.54	–236.72	–24.58
(^{tBu}POCOP)$TiCl_3$ (C_2)	–232.62	56.26	–288.88	–263.33	209.21	–210.77	–24.00

It is noteworthy that the dispersion term remains relatively constant across the whole series, contributing around 25 kcal mol^{-1} to the stability of each complex. The bond energy in **1** between the {$TiCl_3^{+}$} and {$^{tBu}PCP^{-}$} fragments appears to be similar for all symmetries, with values of approximately –250 kcal mol^{-1}. The experimentally observed C_1 symmetric geometry is favoured. The orbital term increases only marginally due to the distortion from C_2 (–223 kcal mol^{-1}) to C_1 (–227 kcal mol^{-1}) symmetry and is in both cases overcompensated by ~5-6 kcal mol^{-1} from the repulsive Pauli term. The overall bond energy seems to be largely governed by

the electrostatic term (which seems unsurprising given the charged fragments), which again is constant for both C_1 and C_2 symmetries of **1**. The energetic penalty from ΔE_{prep} is around 59 kcal mol^{-1} in both cases, which dominantly (~60%) stems from the distortion of the {$TiCl_3^+$} unit (vs. ~40% ligand contribution).

On the other hand, for the (^{tBu}POCOP)$TiCl_3$ complex it is found that the distorted C_1 structure is notably stabilised (~ 7 kcal mol^{-1}) with respect to the C_2-symetric structure. Overall, the binding energies are somewhat lower compared to the ^{tBu}PCP complex (10-18 kcal mol^{-1}). The pronounced distortion towards the crystal structure symmetry (C_1) renders the ΔE_{orb} term the most strongly stabilising (–237 kcal mol^{-1}) compared to the C_2-symmetric structure. Concomitantly, this comes at the cost of 30 kcal mol^{-1} in additional Pauli repulsion that in fact exceeds the energetic gain from the orbital term by ~3 kcal mol^{-1}. The electrostatic term becomes more favourable though and is only balanced by a moderate preparation energy (60.5 kcal mol^{-1}). It is interesting that in case of the distorted C_1 complex **1** (i.e. with analogous structure to the ^{tBu}POCOP ground state) the ΔE_{orb} and ΔE_{Pauli} terms are remarkably similar to the POCOP system, but exactly cancel each other (–237 kcal mol^{-1} and +237 kcal mol^{-1} for ΔE_{orb} and ΔE_{orb}, respectively). However, here the electrostatic term is not significantly affected and remains around the same value as the other symmetries (290 kcal mol^{-1}). It may be speculated that the presence of lone pairs on the oxygen bridges in the ^{tBu}POCOP ligand contributes a repulsive term between the ligand fragment and the chlorides, which can be alleviated through a strong distortion of the metal chloride unit. In **1** this extreme distortion comes at a substantial cost (ΔE_{prep} = 67 kcal mol^{-1}) which in turn balances the interaction energy. Clearly the interactions are very nuanced, and the equilibrium geometries offer the best compromise between steric and electronic factors. The deformation densities from the ETS-NOCV analysis are shown in the Figures below, representing dominantly σ-donations from the ligand to the metal fragment.

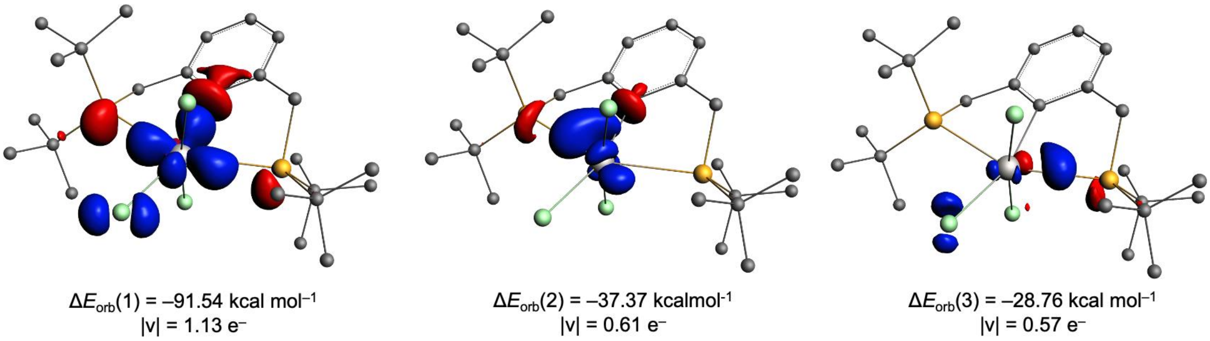

Figure S10: ETS-NOCV (isosurface = 0.005 au) for C_1-symmetric **1**. The direction of the charge flow from fragments to full complex in the deformation densities red → blue.

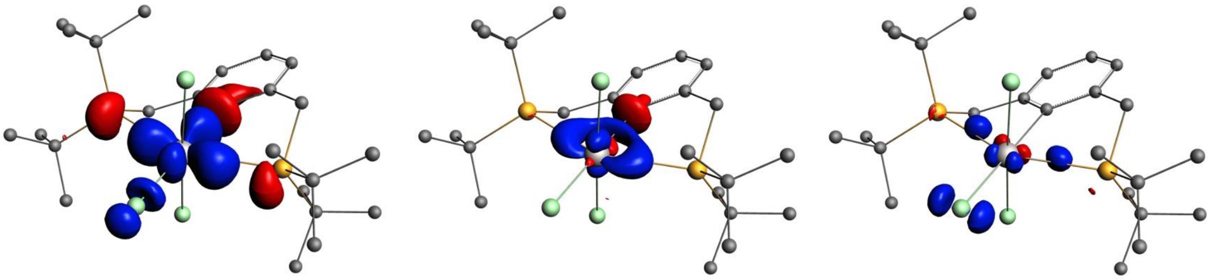

$\Delta E_{orb}(1)$ = –88.64 kcal mol^{-1}
|v| = 1.08 e^{-}

$\Delta E_{orb}(2)$ = –37.97 kcal mol^{-1}
|v| = 0.60 e^{-}

$\Delta E_{orb}(3)$ = –27.41 kcal mol^{-1}
|v| = 0.57 e^{-}

Figure S11: EDA-NOCV (isosurface = 0.005 au) for C_2-symmetric **1**. The direction of the charge flow from fragments to full complex in the deformation densities red → blue.

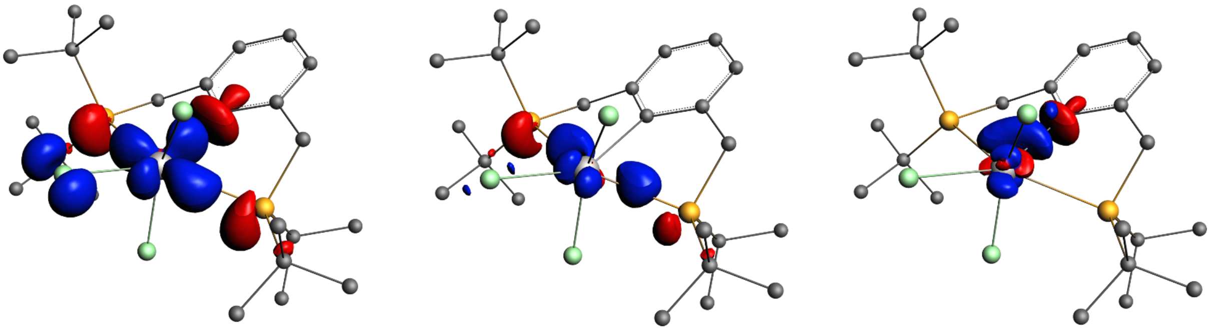

$\Delta E_{orb}(1)$ = –94.41 kcal mol^{-1}
|v| = 1.19 e^{-}

$\Delta E_{orb}(2)$ = –39.59 kcal mol^{-1}
|v| = 0.71 e^{-}

$\Delta E_{orb}(3)$ = -31.75 kcal mol^{-1}
|v| = 0.56 e^{-}

Figure S12: EDA-NOCV (isosurface = 0.005 au) for distorted C_1-symmetric **1**. The direction of the charge flow from fragments to full complex in the deformation densities red → blue.

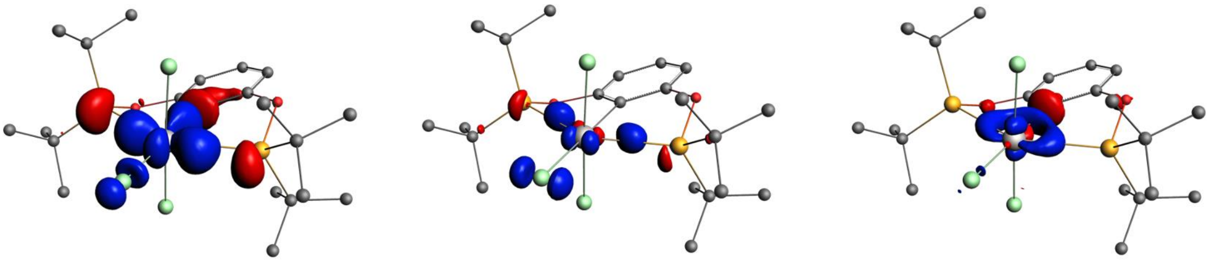

$\Delta E_{orb}(1)$ = –82.80 kcal mol^{-1}
|v| = 1.04 e^{-}

$\Delta E_{orb}(2)$ = –29.83 kcal mol^{-1}
|v| = 0.62 e^{-}

$\Delta E_{orb}(3)$ = –35.28 kcal mol^{-1}
|v| = 0.56 e^{-}

Figure S13: EDA-NOCV (isosurface = 0.005 au) for C_2-symmetric (^{tBu}POCOP)$TiCl_3$**.** The direction of the charge flow from fragments to full complex in the deformation densities red → blue.

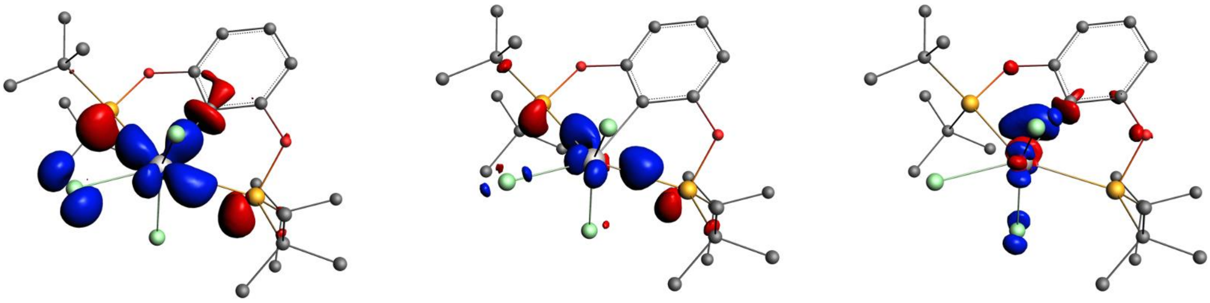

$\Delta E_{orb}(1) = -94.51$ kcal mol^{-1}
$|v| = 1.20$ e^{-}

$\Delta E_{orb}(2) = -40.40$ kcal mol^{-1}
$|v| = 0.71$ e^{-}

$\Delta E_{orb}(3) = -27.92$ kcal mol^{-1}
$|v| = 0.57$ e^{-}

Figure S14: EDA-NOCV (isosurface = 0.005 au) for C_1-symmetric (^{tBu}POCOP)$TiCl_3$**.** The direction of the charge flow from fragments to full complex in the deformation densities red → blue.

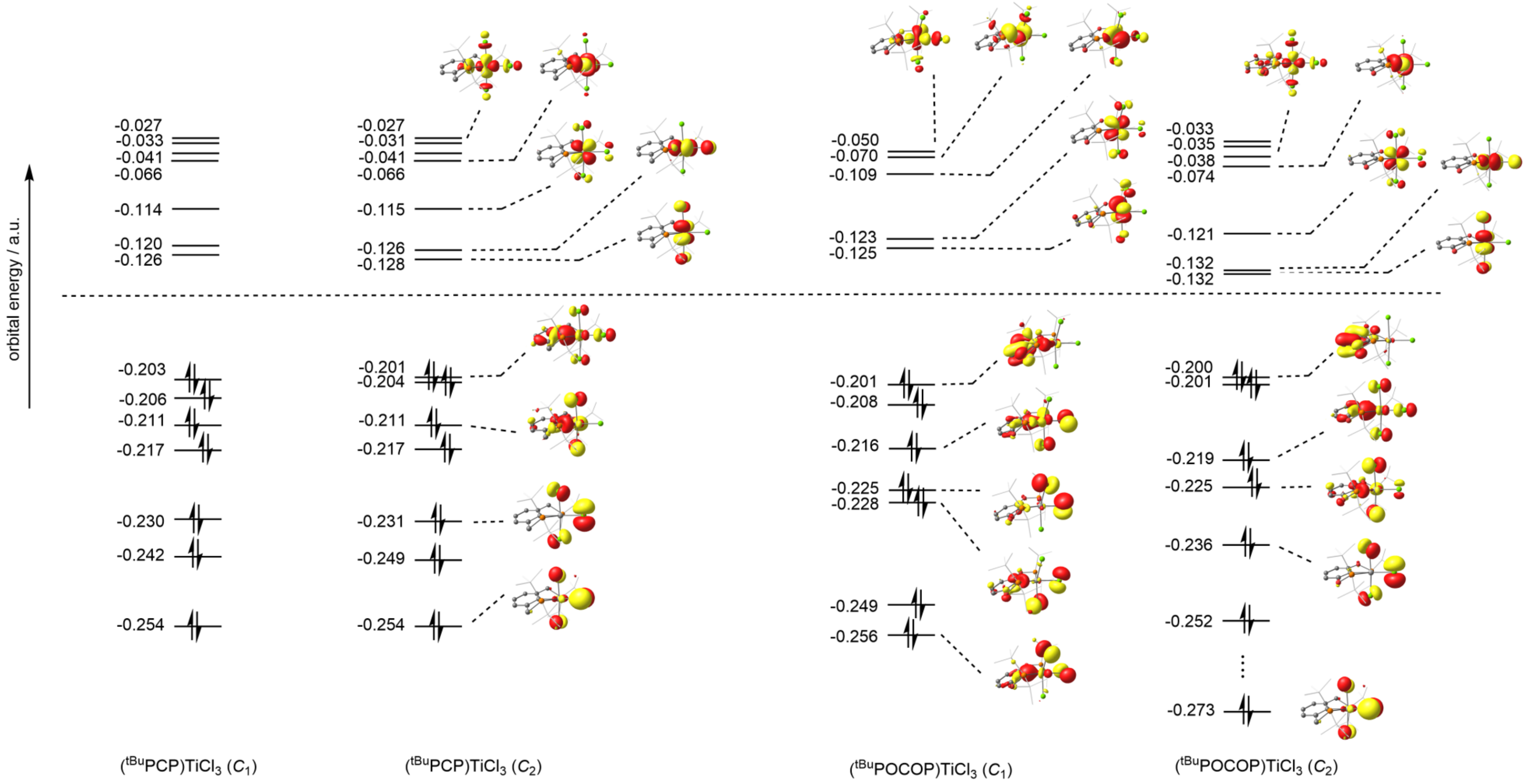

Figure S15: Canonical Kohn-Sham MO diagram for **1** and (^{tBu}POCOP)TiCl$_3$ (isosurface = 0.05 au).

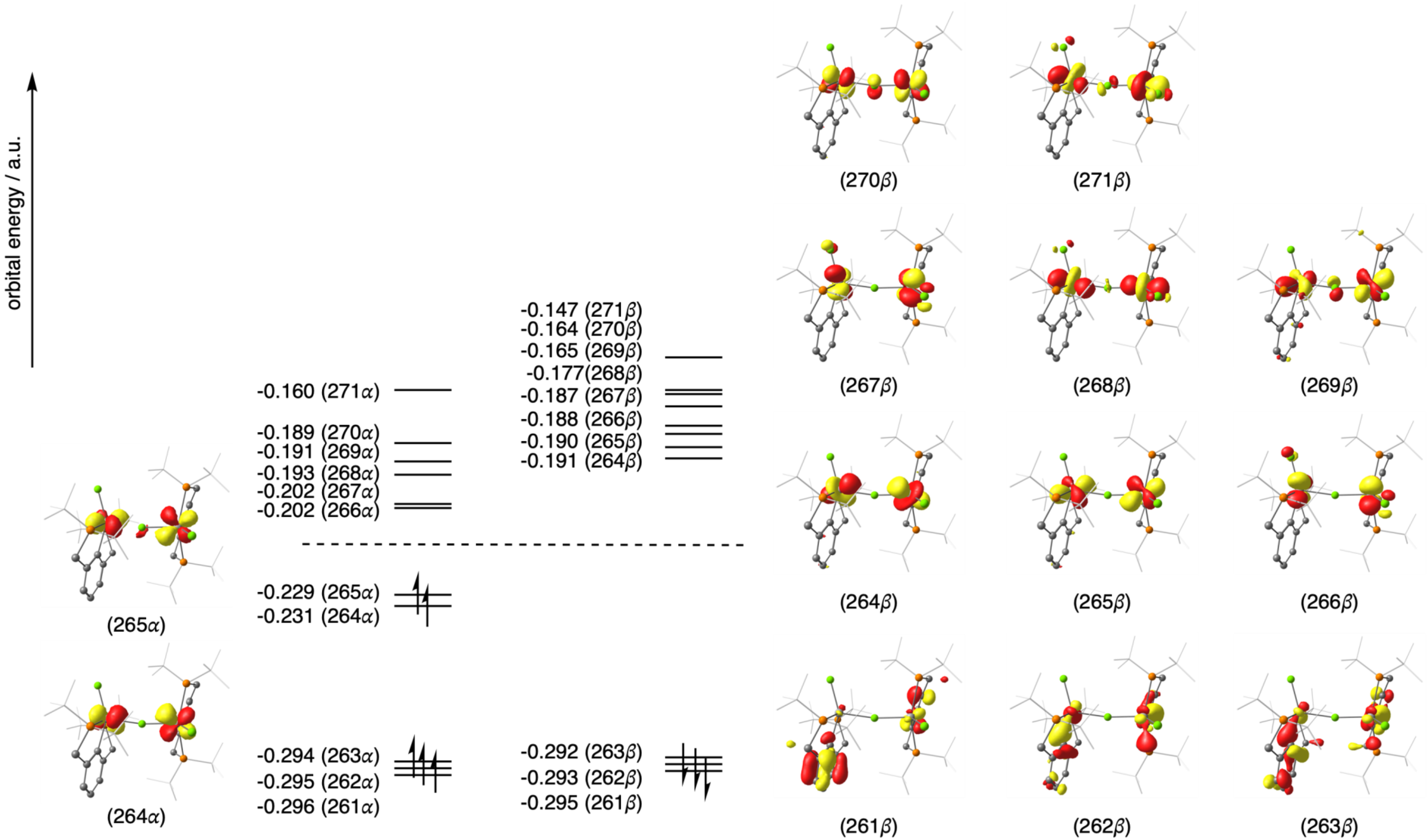

Figure S16: Canonical Kohn-Sham MO diagram for **3** in its triplet (*S* = 1) spin state (isosurface = 0.05 au).

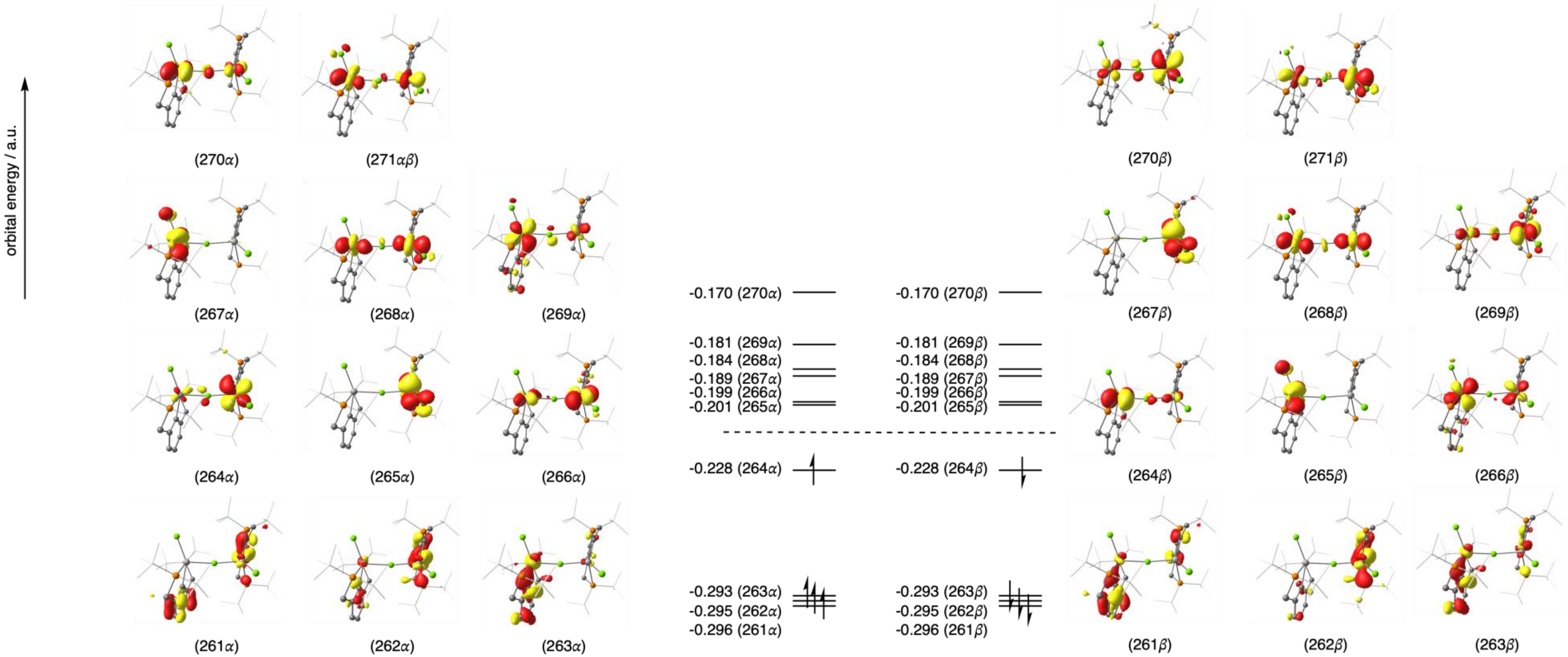

Figure S17: Canonical Kohn-Sham MO diagram for **3** in its broken-symmetry singlet (M_S = 0) spin state (isosurface = 0.05 au).

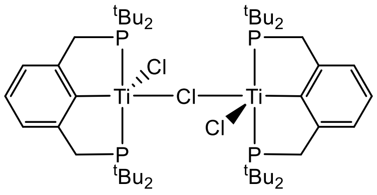

3 (0.0)

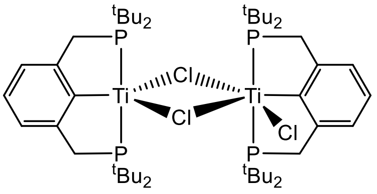

3'
[no minimum]

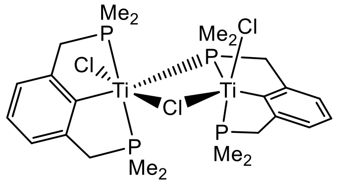

$\mathbf{3}^{\mathbf{Me''}}$ (0.0)

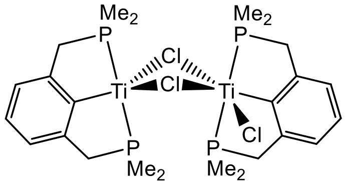

$\mathbf{3}^{\mathbf{Me'}}$ (8.2)

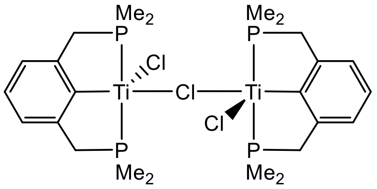

$\mathbf{3}^{\mathbf{Me}}$ (38.0)
[partial optimisation]

Figure S18: Relative electronic energies (kcal mol^{-1}) of structural isomers of **3** (R = ^{t}Bu) and its truncated model $\mathbf{3}^{\mathbf{Me}}$ (R = CH_3) in their triplet spin states (*S* = 1). Energies are given in kcal mol^{-1} (BP86-D3/def2-TZVP/def2-SVP). The bridged Ti(μ_2-Cl)$_2$Ti isomer is not a minimum for **3**, instead the structure converges to the linear Ti–Cl–Ti motif during geometry optimisation.

4. References

[1] Gaussian 16, Revision B.01, M. J. Frisch, G. W. Trucks, H. B. Schlegel, G. E. Scuseria, M. A. Robb, J. R. Cheeseman, G. Scalmani, V. Barone, G. A. Petersson, H. Nakatsuji, X. Li, M. Caricato, A. V. Marenich, J. Bloino, B. G. Janesko, R. Gomperts, B. Mennucci, H. P. Hratchian, J. V. Ortiz, A. F. Izmaylov, J. L. Sonnenberg, D. Williams-Young, F. Ding, F. Lipparini, F. Egidi, J. Goings, B. Peng, A. Petrone, T. Henderson, D. Ranasinghe, V. G. Zakrzewski, J. Gao, N. Rega, G. Zheng, W. Liang, M. Hada, M. Ehara, K. Toyota, R. Fukuda, J. Hasegawa, M. Ishida, T. Nakajima, Y. Honda, O. Kitao, H. Nakai, T. Vreven, K. Throssell, J. A. Montgomery, Jr., J. E. Peralta, F. Ogliaro, M. J. Bearpark, J. J. Heyd, E. N. Brothers, K. N. Kudin, V. N. Staroverov, T. A. Keith, R. Kobayashi, J. Normand, K. Raghavachari, A. P. Rendell, J. C. Burant, S. S. Iyengar, J. Tomasi, M. Cossi, J. M. Millam, M. Klene, C. Adamo, R. Cammi, J. W. Ochterski, R. L. Martin, K. Morokuma, O. Farkas, J. B. Foresman, and D. J. Fox, Gaussian, Inc., Wallingford CT, 2016.

[2] G. te Velde, G.; F. M. Bickelhaupt, E. J. Baerends, C. Fonseca Guerra, S. J. A. van Gisbergen, J. G. Snijders, T. Ziegler, *J. Comp. Chem.*, **2001**, *22*, 931–967.

[3] A. D. Becke, *Phys. Rev. A*, **1988**, *38*, 3098–3100.

[4] J. P. Perdew, *Phys. Rev. B*, **1986**, *33*, 8822–8824.

[5] F. Weigend, R. Ahlrichs, *Phys. Chem. Chem. Phys.*, **2005**, *7*, 3297–3305.

[6] K. Eichkorn, F. Weigend, O. Treutler, R. Ahlrichs, *Theor. Chem. Acc*., **1997**, *97*, 119–124.

[7] S. Grimme, J. Antony, S. Ehrlich, H. Krieg, *J. Chem. Phys.*, **2010**, *132*, 154104.

[8] S. Grimme, S. Ehrlich, L. Goerigk, *J. Comput. Chem*., **2011**, *32*, 1456–1465.

[9] a) A. D. Becke, *J. Chem. Phys.*, **1993**, *98*, 5648–5652. b) C. Lee, W. Yang, R. G. Parr, *Phys. Rev. B*, **1988**, *37*, 785–789. c) S. H. Vosko, L. Wilk, M. Nusair, *Can. J. Phys.*, **1980**, *59*, 1200. d) P. J. Stephens, F. J. Devlin, C. F. Chabalowski, M. J. Frisch, *J. Phys. Chem.*, **1994**, *98*, 11623-11627.

[10] E. Caldeweyher, C. Bannwarth, S. Grimme, *J. Chem. Phys.*, **2017,** *147*, 034112.

[11] E. van Lenthe, E. J. Baerends, *J. Comput. Chem.,* **2003,** *24*, 1142–1156.

[12] P. Mitoraj, A. Michalak, T. Ziegler, *J. Chem. Theory. Comput.,* **2009,** *5*, 962–975.
